# An optic to replace space and its application towards ultra-thin imaging systems

**DOI:** 10.1038/s41467-021-23358-8

**Published:** 2021-06-10

**Authors:** Orad Reshef, Michael P. DelMastro, Katherine K. M. Bearne, Ali H. Alhulaymi, Lambert Giner, Robert W. Boyd, Jeff S. Lundeen

**Affiliations:** 1 Department of Physics, University of Ottawa, Ottawa, ON Canada; 2 School of Electrical Engineering and Computer Science, University of Ottawa, Ottawa, ON Canada; 3 Institute of Optics and Department of Physics and Astronomy, University of Rochester, Rochester, NY USA; 4 Present Address: Département de Physique et d’Astronomie, Université de Moncton, Moncton, NB Canada

**Keywords:** Metamaterials, Imaging and sensing

## Abstract

Centuries of effort to improve imaging has focused on perfecting and combining lenses to obtain better optical performance and new functionalities. The arrival of nanotechnology has brought to this effort engineered surfaces called metalenses, which promise to make imaging devices more compact. However, unaddressed by this promise is the space between the lenses, which is crucial for image formation but takes up by far the most room in imaging systems. Here, we address this issue by presenting the concept of and experimentally demonstrating an optical ‘spaceplate’, an optic that effectively propagates light for a distance that can be considerably longer than the plate thickness. Such an optic would shrink future imaging systems, opening the possibility for ultra-thin monolithic cameras. More broadly, a spaceplate can be applied to miniaturize important devices that implicitly manipulate the spatial profile of light, for example, solar concentrators, collimators for light sources, integrated optical components, and spectrometers.

## Abstract (web summary)

The need for space between lenses in optical systems results in a trade-off between potential for miniaturisation and achieved resolution. Here, the authors demonstrate a device that propagates light longer than its thickness, a spaceplate, and can therefore replace space in optical systems.

## Introduction

Metasurfaces—engineered surfaces consisting of sub-wavelength scatterers—have attracted a great deal of attention for enabling flat optical components^1–6^. These devices have been implemented in a diverse set of novel linear^7–11^ and nonlinear optical^12–14^ applications, including sub-wavelength-scale broadband achromatic lenses^15^, the generation of various transverse spatial modes^1^^,^^8^, lasing^16,17^, polarimetry^18^, and holograms^19^, among others. Notably, metalenses are seen as the most promising by far due to their impact in miniaturizing imaging systems^20,21^. However, in all imaging systems, lenses represent just one of the two main components; the other, sometimes overlooked in this context, is the millimeter-to-meter-scale optical propagation surrounding the lenses and separating them from the object and image. As evidenced by the long physical length of a typical (e.g., Galilean) telescope, the distances between lenses are just as critical to image formation as the lenses themselves, and can easily be greater than the summed thicknesses of the lenses by an order of magnitude. To date, no work has been published that addresses this dominant contribution to the size of many optical systems.

**Fig. 1:**
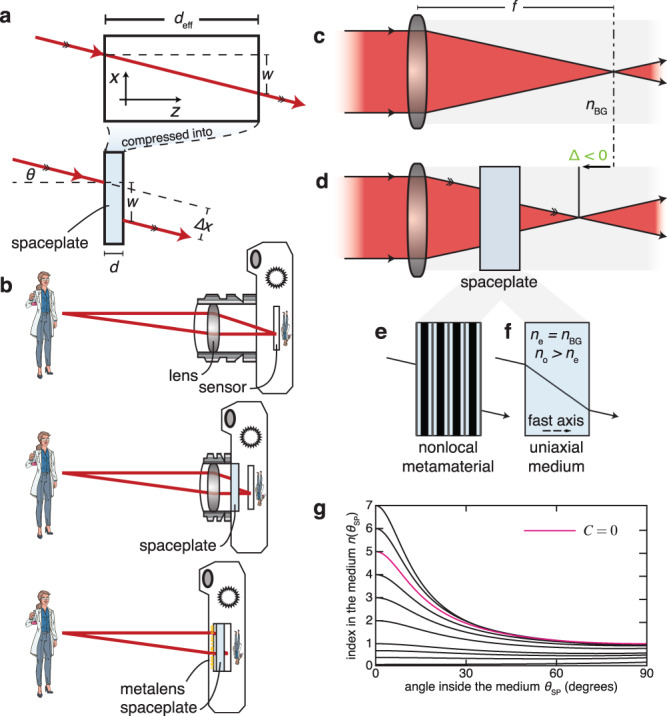
Operating principle of a spaceplate. **a** A spaceplate can compress a propagation length of *d*_eff_ into a thickness *d*. For example, a beam incident on the spaceplate at angle *θ* will emerge at that same angle and be transversely translated by length *w* (resulting in a lateral beam shift Δ*x*), just as it would for *d*_eff_ of free space. **b** Adding a spaceplate to an imaging system such as a standard camera (top) will shorten the camera (center). An ultrathin monolithic imaging system can be formed by integrating a metalens and a spaceplate directly on a sensor (bottom). **c** A lens focuses a collimated beam at a working distance corresponding to its focal length *f*. **d** A spaceplate will act to shorten the distance from the lens to the focus by a distance |Δ|. The emerging rays are parallel to the original incident rays, which preserves the lens strength. The plate therefore effectively propagates light for a longer length than the physical space it occupies. This effect can be achieved using **e**, a nonlocal metamaterial, or **f**, for the extraordinary ray for propagation along the fast axis (e) of a uniaxial birefringent medium with *n*_BG_ = *n*_e_. **g** A spaceplate can be made of a homogeneous medium with any of these angle-dependent refractive index curves, parametrized by the quantity *C*.

We present here a potential path toward replacing these distances with an optical element that we call a “spaceplate”. The functionality of a spaceplate is highlighted in Fig. 1a. This element would occupy a physical thickness of *d*, while propagating light for an effective length of *d*_eff_ *>* *d*, where the ratio between these two quantities *R ≡* *d*_eff_*/d* is the compression factor of the plate. Some metamaterials, such as those based on transformation optics^22,23^, already feature the compression of electromagnetic fields (e.g., for field concentrators^24^ or hyperlenses^25^). Though compression of propagation distance can be implicit in these works, this compression has not been an aim unto itself, particularly for reducing the length of imaging systems. There are many optical devices that implicitly use imaging (e.g., a grating spectrometer works by imaging a slit^26^) or that spatially manipulate light using its propagation, such as solar concentrators^27^, multiplane mode demultiplexers^28^, or multimode interferometers in integrated optics^29^. All of these devices could be shortened through use of a spaceplate, leading to significant practical advantages.

It is easiest to describe the operation of the spaceplate by way of example—to this end, we consider the use of a spaceplate in a camera, as illustrated in Fig. 1b. The space between the lens and the sensor of a camera is dictated to a large degree by the focal length *f* of the lens. A relatively large focal length is necessary to suitably magnify an image, which leads to long lens barrels in cameras. One approach toward reducing this length could be the use of a spaceplate, allowing for the large magnification of a faraway object without the need for a large propagation length. While nominally this is also the goal of a telephoto lens, in practice the length of a telephoto lens barrel has been approximately constrained to be 0.8 times its effective focal length^30^ (see Supplementary Note 2: “Comparison to a telephoto lens” for more discussion). Unlike a telephoto lens, a spaceplate could therefore break the trade-off between lens-barrel length and image magnification. Moreover, since the resulting image may now be large, so can the image sensor (e.g., the charge-coupled device (CCD) array). One can capitalize on this larger sensor by using larger pixels for low-light sensitivity, or a greater number of pixels for a higher resolution. In this way, the spaceplate could one day break the trade-off between camera miniaturization and any of resolution, sensitivity, magnification, or field-of-view.

In this work, we develop the general concept and theory of the spaceplate, followed by the proposal of several physically realizable types of spaceplates. In particular, we introduce and fully simulate the operation of a proof-of-principle multilayer design (i.e., a “thin-film stack”). This 25-layer design consists of only two materials, barely utilizing the full potential of current fabrication capability, which reaches over a thousand layers of multiple different materials^31^. We follow this modeling by some experimental demonstrations of two other types of spaceplates, albeit ones with a modest compression factor. These experiments will establish that spaceplates can be polarization-independent, exhibit broadband operation, or have a large numerical aperture; thus, in concept, spaceplates can satisfy all three of these performance targets. However, it remains to be shown that these targets are compatible with each other or with a usefully large compression factor and effective length *d*_eff._ We conclude with some discussion and analysis of the further advances that are necessary for spaceplates to become practical devices.

## Results

### Fourier optics analysis of the operation of a spaceplate

To define the action of the spaceplate, we make use of Fourier optics^32^. Namely, we consider how free propagation transforms each plane-wave component of an incident field. Each plane wave is a given transverse spatial Fourier component with momentum vector **k**. The amplitude of each *k*-vector component is preserved in its free-space propagation, whereas its phase is shifted. Consider two points along *z* separated by *d*_eff_ for a given plane wave. The wave’s phase difference between these points will be *φ* = *k*_*z*_*d*_eff_, where *k*_*z*_ = |**k**| cos *θ*, and *θ* is the angle of **k** from the *z*-axis. Combining this amplitude and phase behavior, the Fourier transfer function of free space is *H(***k***)* *=* exp*(ik*_*z*_*d*_eff_*)*. Free propagation will effectively multiply each incident plane wave by this factor.

A spaceplate needs to produce the same transfer function. A transfer function *H(*k*)* with the *k*-vector **k** |**k**| = (2π*n*_BG_*/*λ*)* yields a propagation phase of *φ* *=* (2*πn*_BG_*d*_eff_cos *θ/λ*) ≡ *φ*_BG_, where *λ* is the wavelength of light in vacuum and *n*_BG_ is the index of the background medium (BG) in the *d*_eff_ slab of space. The critical action of a spaceplate is thus to produce an angle-dependent phase profile *φ*_SP_ that is equal to *φ*_BG_ (*θ*, *d*_eff_), the phase from propagation through a distance *d*_eff_ of the background medium. However, the spaceplate must do so within a distance shorter than *d*_eff_—in particular, in a plate thickness *d*. Note that the angular phase profile *φ*_SP_(*θ*) possesses the following two properties. The first is that the addition of an arbitrary phase offset *φ*_G_ that is global (i.e., independent of *θ*) will not affect the imaging properties of the system^21^. Second, the image will also not be affected if *φ*_SP_(*θ*) is discontinuous as a function of *θ* with discontinuities of an integer multiple *m* of 2*π*; this type of solution would correspond to the Fourier-space analog of a Fresnel lens^33,34^. These two free parameters hint at the substantial flexibility available to design a spaceplate.

Such a momentum-dependent response, where an optical element acts on the phase or magnitude of the spatial Fourier components of a beam, has been called a “nonlocal” response^35–37^. Specifically, an ideal spaceplate would impart the phase,

1 $$\varphi _{\rm{SP}}(k_x,k_y,d_{\mathrm{eff}})=d_{\mathrm{eff}}{({{|{\mathbf{k}}|}}^2-k_x^2-{k}_{y}^{2})}^{1/2},$$

whereas its “local” position-dependent counterpart is a positive, spherical, thin lens, *φ*_lens_ (*x*, *y*, *f*) = (2*π*/*λ*)(*f*^2^ − *x*^2^ − *y*^2^)^1/2^ (ref. ^38^). Unlike a position-dependent response, a purely momentum-dependent response, such as the one in Eq. (1), cannot redistribute momentum components. That is, it cannot redirect the angle of a light ray and, thus, it comes with no magnification and has no optical power (i.e., dioptric power), unlike curved mirrors or lenses. Therefore, a spaceplate is an optical element complementary to the lens.

Nonlocal response engineering has been a fruitful research direction bearing applications, such as angular pass-filtering^39^, image processing^40–43^, and analog computing^36^. One previous work in nonlocal responses used a lens system similar to a 4f telescope to impart a *k*-dependent phase and magnitude response^36^. However, the use of a lens and propagation to create a spaceplate defeats its purpose of replacing propagation. A metamaterial, on the other hand, has only ever been engineered to have an angle-dependent transmittance^36,39–42^, thereby solely affecting the magnitude of the Fourier component. In contrast, we focus on materials that impart a phase to each Fourier component. In order to achieve this behavior, we consider spaceplate designs that are translationally invariant along the transverse directions *x* and *y*. This invariance guarantees that a transmitted wave will have the same *k*-vector as the incident wave, which is a necessity for unity transmittance, |*H*| = 1. By manipulating the momentum-dependent phase, the spaceplate is a first example along a new avenue in nonlocal metamaterials research.

### A multilayer spaceplate design

Since nonlocal responses are based in momentum space, and not in position space, such as with metasurfaces, it is at first glance not obvious whether a nonlocal response corresponding to a spaceplate may be realized in a physical system, or whether a realistic spaceplate would have any intrinsic trade-offs between its performance parameters. We now explore whether a spaceplate can be designed out of multilayer stack (Fig. 1e). Since this structure is made up of parallel flat layers of various materials, it possesses the transverse translational invariance that we desire. Moreover, the production of these stacks is a mature technology, appearing in many consumer and industrial products, with commercial companies capable of fabricating sophisticated designs with thousands of layers of several different materials. Consequently, such stacks can incorporate considerable complexity and design freedom. In ref. ^36^, multilayer stacks were theoretically considered for general nonlocal responses, and a structure was designed that, in modeling, modulated the momentum-dependent transmittance magnitude. Instead, we design a stack to impart the momentum-dependent phase *φ*_BG_(*θ*, *d*_eff_) that we require for a spaceplate (where *d*_eff_ is greater than *d*, the total stack thickness). The purpose of this design is simply to establish that a multilayer architecture can produce a spaceplate and also to determine some initial performance characteristics.

**Fig. 2:**
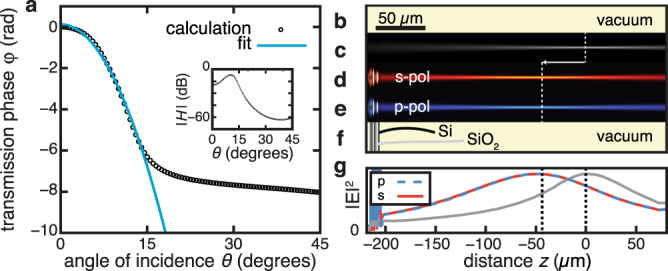
A nonlocal metamaterial spaceplate. **a** A multilayer stack consisting of alternating layers of silicon and silica of various thicknesses is engineered to reproduce the Fourier transfer function *H* for propagation through vacuum for incident angles smaller than *θ* = 15° at an optical wavelength of *λ* = 1550 nm. Plotted is the calculated transmission phase *φ*_SP_ of the metamaterial spaceplate (black circles) and a fitted vacuum transfer function phase *φ*_BG_ (blue curve). Here, we have subtracted a global phase of *φ*_G_ = −0.05 rad. The fitted compression factor is *R* = 4.9. The inset shows the transmission amplitude |*H*|. **c**–**e** Full-wave simulations of the square of the magnitude of the electric field, |*E*|^2^, of a focusing Gaussian beam (waist of 3*λ*, divergence of 6^◦^) propagating in **c**, vacuum (gray), **d** after propagating an s-polarized beam through the metamaterial (red, to scale), and **e** after propagating a p-polarized beam through the metamaterial (blue, to scale). **b**, **f** The physical layouts of the simulations, to scale. i.e., **b** is vacuum and **f** is the spaceplate structure surrounded by vacuum. **g** A cross section of |*E*|^2^ along the beam axis. Transmission through the spaceplate advances the focus position along *z* by Δ = −43.2 µm for both p-polarized (dashed blue) and s-polarized (solid red) light.

Since a deterministic and analytic design method for general multilayer stacks has yet to be invented, we use an optimization-based design method, as in ref. ^36^. In particular, we use a genetic algorithm targeting *φ*_BG_(*θ*) that maximizes the compression factor *R*. To set a realistic but relevant goal, we aim only to produce this phase response for a numerical aperture that matches that of modern smartphone cameras, that is, out to an incident angle of *θ* = 15°, i.e., NA = 0.26 (Fig. 2a). Similarly, in order to aim for an easily fabricated design, we restrict the algorithm to two common materials, silica and silicon. This restriction is in contrast to the work in ref. ^36^, where they employed permittivities of arbitrary values idealized to be lossless. We limited our structure to a total thickness of ~10 µm and a maximum of 40 layers so that the algorithm could comfortably run on a standard personal computer. The algorithm took four hours to yield a *d* *~* 10 µm-thick, 25-layer structure. It acts as a spaceplate with a compression factor of *R* = 4.9 for vacuum-filled space (*n*_BG_ = 1) for 1550 nm wavelength light (see Supplementary Note 3: “Spaceplate metamaterial” for more details on this structure.)

In order to test the performance of the designed multilayer structure, we use full-wave simulations to propagate a converging beam through the stack. Full-wave simulations have been validated in the literature to provide accurate predictions for linear optical responses in dielectric materials, such as the case here^44^. Consequently, we do not fabricate and experimentally test this structure, which, with its moderate value of *R*, small *d*_eff,_ and low transmittance, is still far from being a useful device. Simulations of a focusing beam propagating in vacuum and in a spaceplate are shown in Fig. 2b–f. Analogous to in Fig. 1c, d, these show that this structure indeed advances the beam focus in vacuum toward the plate, as desired. Figure 2g shows the advance is Δ = −43.2 μm, which corresponds to a compression factor of *R* = 5.2, in approximate agreement with the prediction. Thus, 10 μm of spaceplate metamaterial may be used to replace propagation through over 50 μm of the background medium, here, vacuum. Though these properties were not explicitly requested by our optimization algorithm, our simulations show that this device design is both polarization insensitive and advances the focus for a bandwidth spanning 30 nm (see Supplementary Note 3 for more details on the performance of this structure.) Crucially, the compression factor *R* of this structure exceeds the ratio of any of the indices in the spaceplate (n_Si_ = 3.48, n_SiO2_ = 1.45, *n*_vac_ = 1) and, thereby, demonstrates that this ratio does not impose a fundamental limit on *R*. While here we aimed to replace vacuum, in order to achieve a higher numerical aperture for an imaging system, one could instead design the multilayer structure to replace a higher-index background medium since NA = *n*_BG_ sin(*θ*). The success of the relatively simple structure we designed hints at the promise of more complicated multilayer stacks for creating spaceplates with large compression factors.

### Homogeneous media as a spaceplate

A drawback of multilayer stacks is the need for an optimization-based design method, which provides little physical insight into the limitations and operating mechanisms of a spaceplate. For this reason, in this section, we instead consider the possibility of unstructured spaceplates, i.e., homogeneous media. The nonlocal phase response *φ*_BG_ is created by allowing for an angle-dependent refractive index of the media *n*(*θ*), for which we solve to find

2 $$\frac{n({\theta }_{{\rm{SP}}})}{{n}_{{\rm{BG}}}}=\frac{C\pm \sqrt{{C}^{2}+({ {R} }^{2}-{C}^{2})(1+{ {R} }^{2}{\tan }^{2}{\theta }_{{\rm{SP}}})}}{(1+{ {R} }^{2}{\tan }^{2}{\theta }_{{\rm{SP}}})\cos \,{\theta }_{{\rm{SP}}}}$$

where *θ*_SP_ is the *k*-vector angle within the spaceplate medium and *C* = (*φ*_G_ + 2*πm*(*θ*_SP_))/*φ*_BG_(0, *d*) (see Supplementary Note 4: “Homogeneous spaceplate solutions” for details). Such a homogeneous non-isotropic plate acts as a spaceplate with compression factor *R* for a background medium with refractive index *n*_BG_.

We now discuss the requisite index profile in more detail and identify a physically realizable solution. From here on, we assume *m* = 0 for all angles and take the positive root. Since the global phase offset *φ*_G_ is still arbitrary, so is *C*. Thus, *C* parametrizes an infinite family of solutions, some of which are shown in Fig. 1g. One realizable homogeneous solution is with *C* = 0 (Fig. 1f). This solution is related to but is distinct from a solution from transformation optics that compresses the full electromagnetic field^22,23,45^. Other solutions are presented in Supplementary Note 4.2: “Discussion of specific solutions”. Remarkably, we find that the refractive index described by this solution is that of a negative uniaxial birefringent medium (*n*_o_ > *n*_e_ for ordinary (o) and extraordinary (e) polarizations) with *n*_e_ = *n*_BG_ and its *e*-axis along *z* (see Supplementary Note 4 for details). A light field with e-polarization propagating through this medium experiences a compression factor along *z* of *R* = (*n*_o_/*n*_e_) relative to propagation in isotropic medium *n*_BG_. Theoretically, this negative uniaxial medium acts as a perfect spaceplate for all incident angles.

**Fig. 3:**
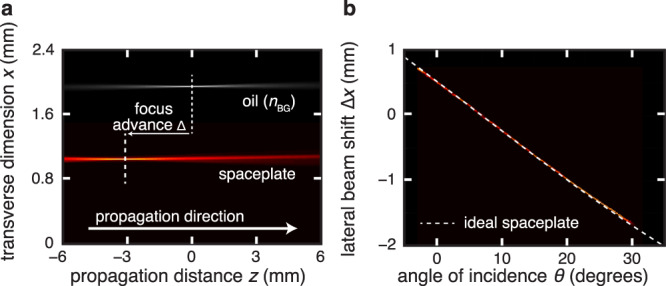
Experimental demonstration of space compression. For all plots, the false color along the plot vertical gives the transverse intensity distribution along *x* at each *z* distance on the horizontal plot axis, with paler color corresponding to higher intensity. **a** Focal shift, Δ = *d* − *d*_eff_. Top data: oil (gray). A converging beam comes to focus in oil at *z* = 0. Bottom data: uniaxial spaceplate (red). Propagation of an e-polarized beam through a calcite crystal with its fast axis along *z* advances the focus position by Δ = −3.4 mm. The corresponding *y* intensity distributions are shown in Supplementary Note 6.1 in the Supplementary Information, demonstrating a fully two-dimensional advance. **b** The walk-off of a beam incident at an angle *θ*. The dashed line gives the lateral beam shift for an ideal spaceplate (i.e., Δ*x* = −(*R* − 1)*d* sin*θ*) with the same thickness *d* and compression factor *R* as the spaceplate in **a**. The uniaxial birefringent crystal acts as a perfect spaceplate for all measured angles of incidence.

In order to show conclusively that the spaceplate concept does work in practice and to explore its limitations, we experimentally test this uniaxial spaceplate. (We present tests of a second type of homogeneous spaceplate, a low-index medium, in Supplementary Note 5: “Low-index spaceplate measurements”.) Naturally available uniaxial crystals have *n*_e_ > 1 and so, instead of comparing to propagation in vacuum, we are limited to comparing to a background medium with *n*_e_ = *n*_BG_, here linseed oil (*n*_BG_ = 1.48). We use a *d* = 29.84-mm-long calcite crystal (CaCO_3_) plate with its optic axis oriented perpendicular to its entrance and exit faces. With *n*_o_ = 1.660 and *n*_e_ = 1.486, the resulting compression factor is a modest *R* = 1.12, far from being of practical use but sufficient for proof-of-principle tests. We propagate a focusing beam through the oil and compare it to the same beam, when propagating through the uniaxial spaceplate placed in oil. An ideal spaceplate will shift this focus by Δ ≡ *d* − *d*_eff_ = −(*R* − 1)*d*. Looking at Fig. 3a, we see that the addition of the spaceplate clearly shifts the focus toward the plate. The measured shift for the e-polarized beam is Δ = −3.4 mm, which agrees well with the predicted shift of Δ = −3.5 mm (see Supplementary Note 6: “Polarization measurements” for details on the o-polarized beam). The spaceplate advances the focus of a beam, just as if it had passed through an additional length of the background medium, thereby showing that the theoretical concept works in practice.

We next experimentally investigate the transverse displacement of a beam incident on a uniaxial spaceplate. This effect is central to the spaceplate’s application, since it also applies to rays in the standard ray-tracing-based design of lens systems. In order to test whether a uniaxial spaceplate is inherently limited in numerical aperture, we vary the angle of incidence *θ* of the beam with respect to the normal of the calcite interface. For each angle, we record the beam’s lateral displacement (indicated by Δ*x* in Fig. 1a) upon exiting, shown in Fig. 3b. The observed displacement Δ*x* (red data) is equal to the ideal displacement of a beam traveling through *d*_eff_ of the linseed oil at angle *θ* (dashed theory curve). Consequently, the uniaxial spaceplate is found to perfectly reproduce the free propagation displacement for all measured angles, i.e., up to *θ* = 35°, corresponding to NA = 0.85 in oil.

**Fig. 4:**
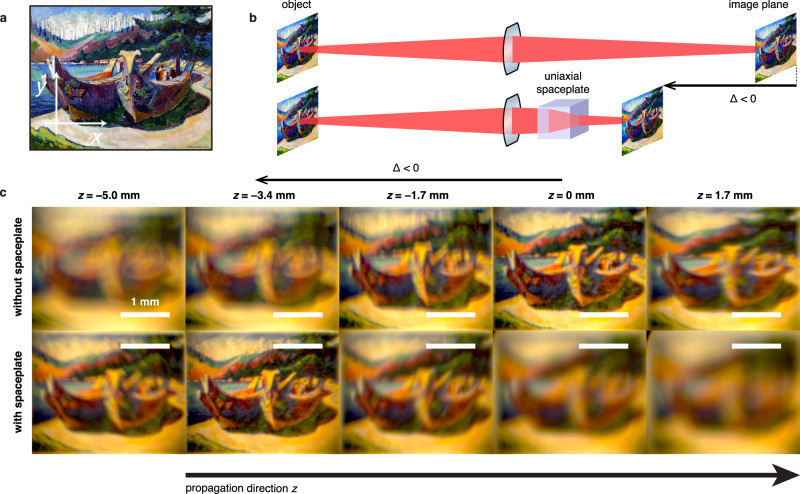
Advance of a broadband visible image using a spaceplate. **a** This print of a painting was illuminated with incoherent white light. **b** An image of the print is formed in either a background medium, glycerol, or through the calcite spaceplate in glycerol. **c** Camera images at various distances *z*. The spaceplate advances the focal plane of the image by Δ = −3.4 mm relative to the glycerol alone. The scale bar is the same length in all the images. This result illustrates that the spaceplate does not change the magnification, i.e., it does not introduce any focusing power. Note that the yellow tint of the recorded images is due to the illumination.

Recently introduced exotic optical material responses, such as negative or near-zero epsilon, have sometimes been associated with material resonances that limit the bandwidth of corresponding devices^46^. To probe whether space compression is an inherently narrowband phenomenon, we test the capability of a spaceplate to reduce the size of a complete full-spectrum visible imaging system. A print of the painting in Fig. 4a is illuminated using an incoherent visible white-light source. A lens system forms an in-focus image of the print at an image plane inside a tank of glycerol placed after the last lens (Fig. 4b). While glycerol (*n*_BG_ = 1.4743) matches *n*_e_ slightly worse than linseed oil, it has a higher transmittivity across the visible spectrum, which makes it more suitable for full-color imaging (see Supplementary Note 1: “Experimental setup”). Figure 4c shows images captured by a CCD camera at a series of different positions *z* along the system axis (see Supplementary Movies 1–3). At *z* = 0, Fig. 4c shows that the captured image is in sharp focus, whereas at position *z* = −3.4 mm, the captured image is still out of focus, as it has not propagated far enough to fully form. We now look at how the spaceplate affects this image formation by placing the calcite crystal into the glycerol before the image plane. The bottom row of Fig. 4c shows the images captured at the same *z* positions as the top row, now with the spaceplate in place. Now, the image comes into focus sooner than with the glycerol alone. Specifically, the captured image is sharp at position *z* = −3.4 mm, whereas in the top row, the image is still forming. Thus, we observe an image advance of Δ = −3.4 mm, in approximate agreement with the theoretical prediction of Δ = −3.5 mm. The entire color image remains in focus simultaneously, illustrating the broadband operation of the uniaxial spaceplate. Furthermore, the magnification of the image is preserved, as evidenced by comparing the sizes of the images at their respective focal planes. Thus, the lens system has been shortened without changing the field of view, the NA, or the magnification. In contrast, shortening the lens system by reducing the lens focal lengths would change all three of these important imaging system parameters.

## Discussion

We have introduced the concept of the spaceplate and presented two types, which we have simulated and experimentally tested, in order to address select potential inherent limitations. The uniaxial spaceplate (and the low-index spaceplate, see Supplementary Note 5) experiments unambiguously demonstrate that a spaceplate is something physically realizable, validating our predictions and Eq. (2). The uniaxial spaceplate experiments, in particular, also show that a spaceplate can be simultaneously broadband in the visible regime, achromatic, have a high NA, as well as high transmission efficiency, albeit for a small compression factor (*R* = 1.12). The metamaterial and low-index spaceplate show that a spaceplate may be polarization-independent. Furthermore, the metamaterial spaceplate shows that a spaceplate may have a compression factor that is many times larger than unity (*R* ≈ 5) and not bounded by the ratio of the indices of any of its constituent materials. It remains to be established whether these properties could be combined in a single spaceplate design that has a usefully large compression factor *R*, similar to what has been accomplished over the past decade in metalenses^1,15,38,47–50^.

In order to gauge the amount of spaceplate improvement that is yet required, we estimate some performance parameters for a few potential applications. We will assume the use of a multilayer spaceplate and, thus, a total thickness *d* of 100 µm, the limit of current thin-film coating technology. The first application is inside a modern smartphone camera, which contains typical spacings of *d*_eff_ = 1–4 mm. A corresponding *R* of 10–40, only a factor of two to eight more than what we have presented, would compress these distances to *d* = 100 µm, inconsequentially small for a camera. Such a spaceplate would need to operate over the full visible wavelength range, have an NA of 0.2, and be polarization insensitive. All of these properties have been demonstrated in the present work, albeit separately. Moreover, sequentially depositing such a spaceplate and a metalens on top of an image sensor would compress an entire camera into an ultrathin monolithic form factor. A second application is to shorten the *d*_eff_ = 2–4 cm distance between the lens and display in virtual reality headsets. This distance has been identified by industry as a key obstacle to adoption^51^. An *R* of 200–400 (for *d* = 100 µm) would effectively eliminate this distance. While the required NA is high (e.g., >0.6, but still below that of our uniaxial spaceplate), other requirements are relaxed. In particular, since liquid crystal displays emit only a single polarization and need only emit three narrowband colors (RGB, such as in laser-based displays), the spaceplate need only function correctly for a single polarization and three optical wavelengths. Lastly, in integrated optics, narrowband (i.e., single wavelength, 1550 nm) single-polarization devices are common^29,52^. Such a spaceplate, with a modest compression factor of *R* = 5, similar to our multilayer design, would significantly increase the density of devices on a chip. Although improvement in spaceplate performance will be necessary, the requirements of these potential applications suggest that these advances are not so large as to be implausible.

We now consider whether and by what means the compression ratio *R* could be improved. As our metamaterial design is based on optimization methods, it cannot directly inform us on whether or not fundamental physics may impose limits on *R*. However, causality does not seem likely to constraint *R*, since the overall time delay for the imaging light to pass through the spaceplate is unconstrained (see Supplementary Note 7: “Implications of causality” for further discussion). Moreover, temporal effects, such as frequency chirp, are free to occur since they are irrelevant to imaging, which is a quasi-static process. Therefore, we do not foresee any fundamental limits to *R*; however, there may exist fundamental trade-offs between *R* and other performance parameters (e.g., operation bandwidth or numerical aperture). Establishing the exact nature and subsequent consequences of these potential trade-offs is a topic for future work.

We propose the following avenues by which the compression factor *R* could be increased. First, the feasibility of two of the homogeneous spaceplate solutions among the set given by Eq. (2) suggests that other solutions in the set may be physically realizable and might bare a higher *R* (see Supplementary Note 4.2 for a discussion of other solutions). Second, industry has sophisticated design methods that could optimize a multilayer spaceplate made of a stack of thousands of layers of multiple materials, which might yield greatly higher values of *R*. A third avenue is engineering an artificial uniaxial medium analogous to the calcite we used. For example, composite materials can be used to create record-breaking anisotropic responses^53^. Or, as another example, a uniaxial medium can be fabricated by alternating sub-wavelength-thick layers between two materials^54–57^. The latter would create a uniaxial spaceplate with the potential advantages that the birefringence (and, hence, *R*) can be larger, broadband, and also slowly varied along *z* to avoid reflection at the interfaces. By following these avenues and others, we expect spaceplates to rapidly improve.

From a broader perspective, the spaceplate further demonstrates the power of nonlocal optical elements that operate directly on the phase of transverse Fourier components of a light field. To the best of our knowledge, this work is the first to design a metamaterial that directly manipulates the phase in *k*-space. Achieving full nonlocal control (e.g., combining the control of transmittance with phase control) would enable all of the benefits of Fourier optics (e.g., spatial filtering) without needing a lens system to access the farfield. In turn, repeatedly iterating between this momentum-dependent Fourier control and position-dependent control has been shown to enable fully arbitrary and lossless spatial transformations of light fields^58^. Using nonlocal metamaterials and local metasurfaces to respectively accomplish these two controls opens the possibility of complete spatial control of light in a monolithic device.

## Methods

### Homogeneous spaceplates

#### Background medium

The background medium for measurements in Fig. 3 is linseed oil (also known as flaxseed oil). This oil (refractive index *n*_BG_ = 1.4795 at an optical wavelength of *λ* = 532 nm) was chosen to match to *n*_e_ of the uniaxial spaceplate material (*n*_e_ = 1.486). The color imaging measurements in Fig. 4 instead used a background medium of glycerol (*n*_BG_ = 1.4743 at 532 nm). While glycerol matches *n*_e_ slightly worse than linseed oil, it has a higher transmittivity across the visible spectrum, which makes it appropriate for full-color imaging.

#### Uniaxial spaceplate

We use a 20.04 mm × 19.98 mm × 29.84 mm (width × height × depth, ±0.06 mm) right rectangular prism made of calcite that was cut with its extraordinary optical axis along the depth direction. The surfaces perpendicular to this axis are polished and used as the entrance and exit faces. Note that the surface quality is low, which somewhat distorts and scatters the beam in the measurements in Fig. 3 (see Supplementary Note 8: “Fabricated spaceplates”). Calcite is negative uniaxial with refractive indices *n*_e_ = 1.486, *n*_o_ = 1.660 at a wavelength of *λ* = 532 nm. For e-polarized light in a background medium with *n*_BG_ = *n*_e_, this crystal gives an expected enhancement factor of *R* = *n*_o_/*n*_e_ = 1.117 and an advance Δ = (1 − R)*d* = −3.494 mm (i.e., a shift toward the crystal). The o-polarized light will experience a medium of isotropic refractive index *n*_o_. Consequently, *R* = *n*_BG_/*n*_o_ = 0.895 and Δ = (1 −*R*)*d* = 3.126 mm.

#### Low-index spaceplate

Our implementation of a low-index spaceplate consists of a glass-faced cylindrical cell containing air (length *d* = 4.37 ± 0.06 mm and diameter = 25.82 ± 0.06 mm). The faces are 0.14 ± 0.01 mm thick microscope coverglass pieces (see Supplementary Note 8).

### Experimental setup

The experimental setup is shown in Supplementary Fig. 1.

#### Light sources

The measurements in Fig. 3 used a 4.5 mW diode laser with an optical wavelength of 532 nm. We spatially filter the laser beam with a single-mode fiber. The beam is then focused to a waist of *ω*_0_ = 9.5 ± 0.5 μm (*e*^−2^ radius) with a 0.69° ± 0.03° *e*^−2^ half-angle and a Rayleigh range of 0.79 ± 0.08 mm, all in the background oil medium. The measurements in Fig. 4 instead used incoherent visible white-light illumination.

#### Field relay system

Both the beam measurements and imaging measurements use a field relay lens system to relay the full *E*_BG_(*x*,*y*,*z*) electric field profile to a region outside the tank containing the background medium and spaceplate. The *f*_1_ = 100 mm lens after the tank, and the *f*_2_ = 200 mm lens are separated by a distance *s*_4F2_ = 300 mm, which constitutes a common lens system known as a 4f system. The resulting magnification is *M* = *f*_2_/*f*_1_ = 2. The system relays the field outside *E*_out_(*x*, *y*, *z*) such that *E*_out_(*Mx*, *My*, *M*_2_*z*/*n*_BG_) ∝ *E*_BG_(*x*, *y*, *z*). Outside the tank, we use an image sensor (CCD) to record the intensity spatial distribution in the *x*,*y* plane. We then scan the CCD along *z*. Five images are taken at each step and averaged to reduce camera noise. A shutter is closed in order to acquire background images, which are subtracted from the raw images to compensate for stray light and camera noise. For the measurements in Fig. 3, we use a monochromatic camera (3088 × 2076 pixels, 2.4 μm × 2.4 μm each, 12 bit). For the measurements in Fig. 4, we use a color camera (1936 × 1216 pixels, 5.86 μm × 5.86 μm each, 12 bit). In the figures, we report the dimensions of the field inside the oil.

#### Beam measurements

The beam measurement setup is shown in Supplementary Fig. 1a. A diode laser produces a beam of wavelength 532 nm with a power of 4.5 mW. This beam is attenuated using a filter and then has its spatial mode filtered by a single-mode fiber. The beam exiting the fiber is collimated. A half-waveplate (λ/2) and polarizing beamsplitter (PBS) are used to vary the beam intensity and polarize the beam. The beam’s polarization is subsequently controlled by a zero-order half-waveplate (λ/2) and quarter-waveplate (λ/4). The lens before the tank, *f*_1_ = 100 mm, is used to focus the beam through the spaceplate. The spaceplate’s entrance surface is located 80 mm from this *f*_1_ lens. The tank contains linseed oil as a background medium. We then use the field relay system to image the transmitted beam. For the measurements in Fig. 3a, we move the camera along *z*, recording an image at steps of 0.02 in (0.508 mm) over a range of 60 mm. For the measurements in Fig. 3b, the camera *z*-position is set so that the camera images the beam focus. In order to measure the lateral beam displacement Δ*x*, the spaceplate is then tilted by an angle *θ* about *y* in steps of 0.25° over a range of 40° and 43.5° for the calcite and air plates, respectively (note that these ranges are the maximum allowed by the clear aperture of the respective spaceplate). For each camera position *z* or crystal angle *θ*, the recorded image is summed along the *y*-direction to arrive at an intensity distribution along *x*. These *x* intensity distributions are presented along the vertical direction of the plots in Fig. 3.

#### Imaging measurements

The imaging measurement setup is shown in Supplementary Fig. 1b. With visible white light, we illuminate a 15 mm × 12 mm print of a painting (First Nations War Canoes in Alert Bay by Emily Carr, 1912) printed on ordinary white paper. At a distance *s*_*oi*_ = 475 mm from the print is a lens of focal length *f*_*i*_ = 500 mm. A further *s*_*i*__1_ = 355 mm from the *f*_*i*_ lens is the first *f*_1_ = 100 mm lens. Together this lens pair (NA = 0.025) collects the the light reflected from the print, transmits the light through the spaceplate in the tank, which then forms in the background medium an image of the print with magnification 0.209 ± 0.001. Between this lens pair is a linear film polarizer (visible broadband, 400–700 nm), which we rotate to set the polarization of the light. The spaceplate is placed *s*_SP_ ≈ 80 mm after this *f*_1_ lens. The background medium in the tank is now glycerol rather than linseed oil since the former has a high transmission across the visible spectrum, which is ideal for full-color imaging. We then use the field relay system to image the field after the spaceplate at various propagation distances *z*. For the measurements in Fig. 4, we move the camera along *z*, recording an image at steps of 0.02 in (0.508 mm) over a range of 100 mm. In the focused image plane, the image has *x* × *y* dimensions of 6.25 mm × 5.03 mm on the camera sensor.

#### Polarization control

A uniaxial spaceplate acts to replace space for e-polarized light. However, the e-polarization direction varies depending on the angle of the incident wavevector relative to the crystal’s extraordinary optic axis. In order for the incident light field to be simultaneously e-polarized and approximately uniformly polarized along one direction, the crystal is tilted slightly about *y* by an angle *α* relative to the incident beam (and system axis). The tilt is *α* = 4.5° for Fig. 3a and *α* = 8° for Fig. 4. An *x*-polarized light field will then be e-polarized with respect to the crystal; a *y*-polarized field will be o-polarized. For the laser, the incident polarization is set by a PBS followed by waveplates. The polarization of the white light is set by a film polarizer designed for broadband visible light. More generally, the uniaxial spaceplate works for a lightfield with an angularly nonuniform polarization that is extraordinary everywhere (e.g., a radially polarized field).

#### Coordinate system

We use *x* × *y* × *z* as a coordinate system for the experiment, where *x* and *y* are the transverse directions and *z* is the optical system axis (i.e., the beam axis). The crystal’s height dimension is along *y*. The extraordinary optical axis of the tilted crystal defines *z*′ of a second coordinate system, *x*′ × *y* × *z*′. Thus, the second coordinate system is related to the first by a rotation about *y* by angle α. The uniaxial spaceplate always acts as *d*_eff_ = *Rd* distance along *z*′ with *R* = *n*_o_/*n*_e_. This tilt reduces the effective distance along *z* by a factor cos *α*, which for small *α* is approximately unity.

### Multilayer metamaterial spaceplate

#### Metamaterial structure

We consider structures made up of planar layers alternating between two materials, silicon and silica (i.e., a “multilayer stack”). Each layer can have an arbitrary thickness larger than 10 nm, set by feasible fabrication capabilities. The combined thickness of the entire stack is designed to be ~10 μm.

#### Genetic algorithm

Our aim is to design a multilayer stack to replace a background medium of vacuum. To do so, we search for a structure that gives a phase profile φ_SP_ that matches the phase profile φ_BG_(*d*_eff_), resulting from propagation through a slab of vacuum of length *d*_eff._ We restrict this aim to a range of incident angle from zero to θ_max_ (i.e., the NA of the spaceplate). The search is conducted with a genetic algorithm whose goals are to maximize *d*_eff_, while minimizing any optical aberration resulting from a nonideal phase profile. To quantify the latter goal, we first calculate the difference of the slope from that of the ideal profile, Δφ′ = φ′_SP_ − φ′_BG_, where φ′ =  ∂φ/∂θ. This angular slope is the relevant quantity to consider since any global phase φ_G_ and phase wraps 2πm will be eliminated by the derivative.

We then find the root-mean-square (RMS) of this difference, Δ*φ'*_RMS_. The RMS deviation Δ*φ'*_RMS_ is an optical aberration that results in an increased beam waist *ω*_SP_ = *ω*_0_(1 + *θ*_max_Δ*φ'*_RMS_) relative to the waist *ω*_0_ = *λ/*(*πθ*_max_) in the absence of the multilayer stack. As a worst-case scenario, this larger waist will increase the Rayleigh range to *z*_SP_ = *πω*^2^_SP_*/λ*. The parameter *z*_SP_ increases with aberration and the inverse of the usable angle *θ*^ − 1^_max_. The two goals of the algorithm can be combined in a single fitness function, *F* = *d*_eff_*/z*_SP_ = *πd*_eff_*θ*^2^_max_*/*(*λ*(1 + *θ*_max_Δ*φ'*_RMS_)^2^), where we have used the small-angle approximation repeatedly. The larger the value of *F* is, the better the performance of the multilayer spaceplate will be.

We now outline the functioning of the genetic algorithm. Each generation in the genetic algorithm had a population size of 500. The DNA of each population member was the material and the thickness of each layer in the stack. We used two materials, silica and silicon. The maximum number of layers was set to 40 and each layer was constrained to have a thickness >10 nm. For each member, we use the standard transfer matrix formalism to calculate the complex transmission amplitude *H* = |*H*|exp(*iφ*_SP_) of the multilayer stack for a set of incident angles *θ*. We use nonlinear regression to fit *φ*_SP_ with an ideal phase profile *φ*_BG_(*d*_eff_)_,_ giving *d*_eff_ and, with this fit, we numerically calculate *φ*'_RMS_.Both the fit and calculation are conducted over a range of input angles from zero to *θ*_max_ = 15°. With these performance parameters, we find the fitness *F* of each population member. The device thickness of the first generation is constrained to 10 µm, but this parameter is not constrained for later generations. The algorithm was carried out until there was a convergence in the fitness of the “best” member of each generation. For the structure reported here, this took 4000 generations.

#### Full-wave simulations

The simulation in Fig. 2 was performed using a commercial 2D finite-difference time-domain solver. The boundary conditions are perfectly matched layers. Exact details about the geometry of the structure and material parameters can be found in Supplementary Note 3.

## Supplementary information

Supplementary Info

Description of Additional Supplementary Files

Supplementary Movie 1

Supplementary Movie 2

Supplementary Movie 3

## Data Availability

The data that support the plots within this paper and other findings of this study are available from the corresponding author upon reasonable request.

## Code availability

The code used to analyze the data and the related simulation files are available from the corresponding author upon reasonable request.
