# Supplementary material for: An optic to replace space and its application towards ultra-thin imaging systems
Source: Nat Commun. 2021 Jun 10;12:3512. doi: 10.1038/s41467-021-23358-8 (PMC8192919; doi:10.1038/s41467-021-23358-8)
Supplement: Supplementary file 1 — Supplementary Info [file 41467_2021_23358_MOESM1_ESM.pdf]

Supplementary Information

Below are the Supplementary Notes for *An optic to replace space and its application towards ultra-thin imaging systems* by Orad Reshef, Michael P. DelMastro, Katherine K. M. Bearne, Ali H. Alhulaymi, Lambert Giner, Robert W. Boyd, and Jeff S. Lundeen. In Supplementary Note 1 we have a figure depicting the experimental setup. In Supplementary Note 2, we compare the operation of a spaceplate to that of a telephoto lens. Supplementary Note 3 summarizes details about the nonlocal metamaterial spaceplate structure. In Supplementary Note 4, we derive the refractive index for an anisotropic homogeneous spaceplate medium. We then step through the different types of solutions that are yielded by this derivation and their properties. In Supplementary Note 5 we present the low-index spaceplate, including experimental validation. In Supplementary Note 6, we describe ordinarily and extraordinarily polarized beam measurements conducted with the uniaxial spaceplate, including additional beam-focus measurements as proof of the two-dimensional action of the spaceplate. In Supplementary Note 7, we have included a discussion on the implications of causality on the inherent limitations of a spaceplate. In Supplementary Note 8, we describe the fabricated spaceplates we use in our measurements. Finally, in Supplementary Note 9, we derive the lateral shift Δx.

1 Experimental Setup

Depicted in Supp. Fig. 1 are the setups for the beam measurements (Supp. Fig. 1a) and the imaging measurements (Supp. Fig. 1b). Details can be found in the Methods section.

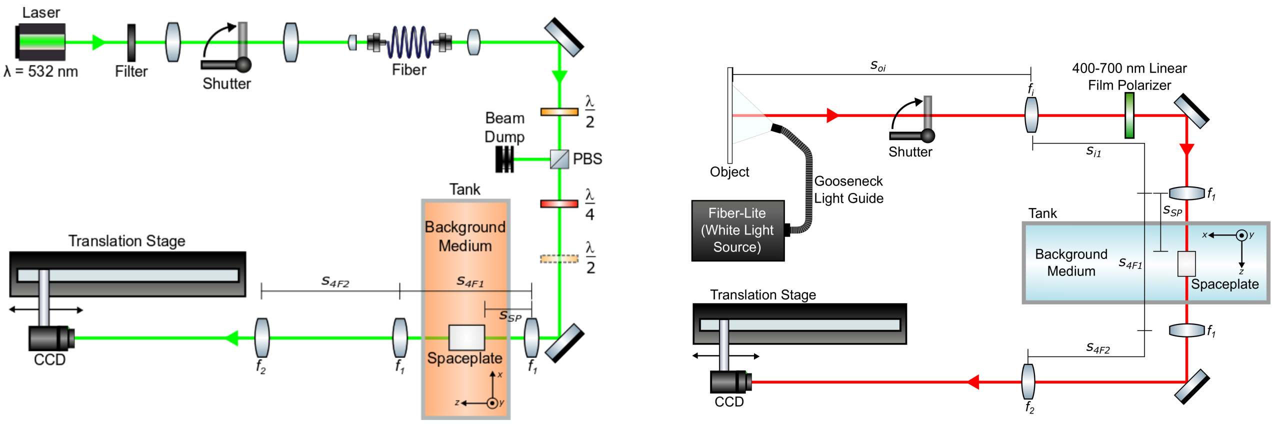

(a) (b)

Supplementary Figure 1 | Experimental setup to measure the action of a spaceplate. See the text for details. **a,** We focus a beam through a spaceplate and measure its shift in z and x. **b,** We perform full colour imaging through a spaceplate.

2 Comparison to a telephoto lens

We compare the properties of a spaceplate with another familiar optical element that is used to reduce the size of an imaging systems— a telephoto lens. A traditional telephoto lens comprises two components: a converging (positive) lens and a diverging (negative) lens, separated by a distance d (Supp. Fig. 2). This combined system decouples the effective focal length from the working distance or the back focal length of the lens. There are established methods for engineering a lens system to exhibit a given effective focal length, with the ratio between the entire track length L and the effective focal length f_{eff} defined

as the telephoto ratio $k = L/f_{\text{eff}}$. It is in principle possible to obtain any telephoto ratio if lenses can be aberration-free, flat and thin, and have any focal length and diameter. However, practical considerations (such as the minimum focal length and alignment between lenses) limit the practical values of $k \sim 0.8$[1].

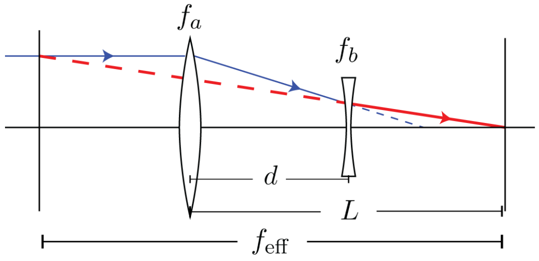

Supplementary Figure 2 | A telephoto lens.

A second point to consider is that the propagation d between the two lenses is critical to the operation of a telephoto; therefore, even for a specific telephoto lens with a vanishing working distance, this required propagation length imposes a minimum size on the system. The spaceplate concept provides an avenue with which one may completely eliminate this space. In fact, a spaceplate could be included within a telephoto lens to reduce its size, for example.

Finally, we note that though a system comprising both a lens and a spaceplate performs a function similar to a telephoto lens, the spaceplate on its own performs a unique function. In particular, as opposed to a telephoto lens, which needs to be designed with *a priori* knowledge of all of the focal lengths, a spaceplate could be added after the fact. It could also be used on its own in other applications that do not fit a telephoto lens system to reduce arbitrary propagation lengths, as it has no lens power and therefore adds no undesired magnification to the system.

3 Spaceplate metamaterial

The metamaterial is designed for operation at an optical wavelength of $\lambda = 1550$ nm. At this wavelength, the complex refractive index of silicon is $n_{\text{Si}} = 3.48985 + 0.00982674i$, and the refractive index of silica glass is $n_{\text{SiO}_2} = 1.45611$. The device has 25 layers and a total thickness of 10.1752 µm. Supplementary Table 1 lists the individual layer thicknesses; Supplementary Figure 3 depicts the cross-section of the metamaterial to scale.

The values in Supplementary Table 1 are listed up to 3 decimal places, as was produced by the genetic algorithm. As can be inferred by the convergence test shown in Supp. Fig. 5, the device is robust to fabrication imperfections, and so this level of precision is not necessary to generate a metamaterial spaceplate that demonstrates a focus advance.

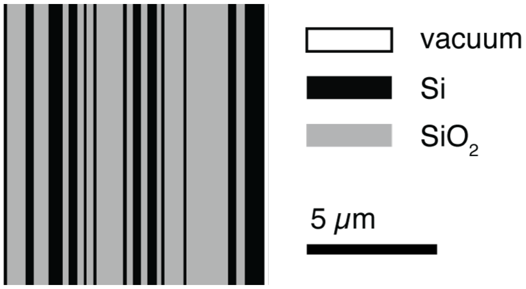

Supplementary Figure 3 | Schematic of the nonlocal metamaterial spaceplate. The spaceplate consists of a multilayer stack formed of two materials, silicon (Si) and silicon dioxide (SiO_2). The schematic is to scale for the given scalebar.

Layer	Material	Thickness (nm)
1	Si	133.86
2	SiO_2	722.324
3	Si	319.406
4	SiO_2	573.293
5	Si	551.083
6	SiO_2	232.074
7	Si	340.955
8	SiO_2	254.686
9	Si	105.252

Layer	Material	Thickness (nm)
10	SiO_2	265.555
11	Si	124.592
12	SiO_2	1032.82
13	Si	145.7
14	SiO_2	239.521
15	Si	313.439
16	SiO_2	252.054
17	Si	371.439

Layer	Material	Thickness (nm)
18	SiO_2	168.605
19	Si	125.935
20	SiO_2	747.517
21	Si	105.681
22	SiO_2	1629.52
23	Si	318.601
24	SiO_2	334.673
25	Si	766.62

Supplementary Table 1 | Materials and thicknesses of individual layers in the metamaterial spaceplate shown in Supp. Fig. 3.

Supplementary Figure 4 demonstrates the performance of the metamaterial spaceplate for both p-polarized and s-polarized focusing Gaussian beams for operating wavelengths ranging from 1530 nm to 1560 nm.

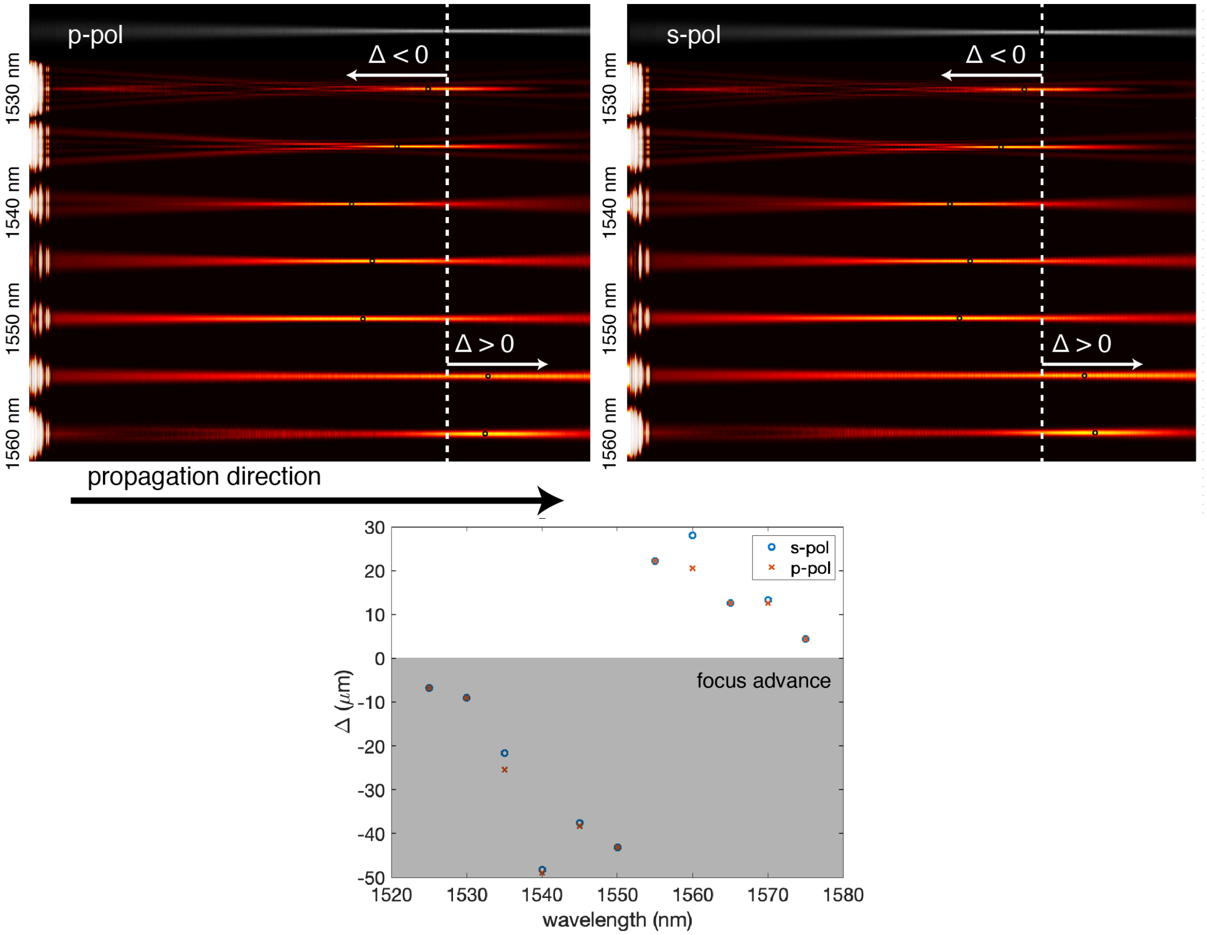

Supplementary Figure 4 | Broadband operation of the metamaterial spaceplate. Top, full-wave simulations of the magnitude of the electric field $|E|^2$ of a focusing Gaussian beam propagating in vacuum (grey, top row) and after propagating through the metamaterial for a range of operating wavelengths (red, all rows but the top row). The device generates a focus advance ($\Delta < 0$) for both polarizations and for wavelengths ranging from 1530 – 1560 nm. **Bottom,** the total focus advance Δ as a function of wavelength for this device.

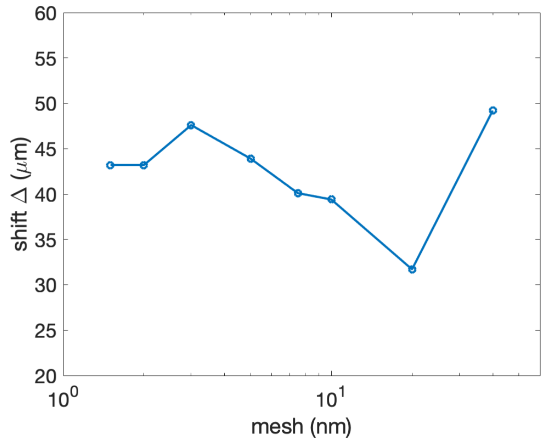

Supplementary Figure 5 | Mesh convergence test for the finite-difference time-domain simulation. The shift of the focus Δ relative to vacuum is simulated as a function of the smallest mesh size along the direction of propagation within the metamaterial. Here, the smallest mesh used was 2 nm. Critically, all these simulations show a sizeable negative shift and, thus, exhibit the spaceplate effect.

4 Homogeneous spaceplate solutions

4.1 General solution for an angle-dependent refractive index

The goal of a spaceplate is to transform an incoming light-field in an identical manner (for the purposes of imaging) to propagation through a slab of an isotropic homogeneous medium. To do so, the spaceplate must multiply the complex amplitude of each plane-wave in an incoming field by the function, $\exp(i\phi_{\text{BG}})$. In particular, a spaceplate of thickness d must impart a phase ϕ_{SP} that is equal to the phase ϕ_{BG} due to the propagation through distance d_{eff} of the medium. Both d and d_{eff} are along what we set to be the z-axis.

In more detail, in a medium of index n_{BG}, a plane-wave has phase difference, $\phi_{\text{BG}} = \mathbf{k}^{(\text{BG})} \cdot \mathbf{r}$, between two positions separated by vector $\mathbf{r} = (x, y, z = d_{\text{eff}})$, where $\mathbf{k}^{(\text{BG})} = (k_x^{(\text{BG})}, k_y^{(\text{BG})}, k_z^{(\text{BG})})$ is the wavevector. Here, $|\mathbf{k}^{(\text{BG})}| = n_{\text{BG}} k_0 \equiv k_{\text{BG}}$ is the wavenumber in the medium and $k_0 = 2\pi/\lambda$ is the vacuum wavenumber. Inside the spaceplate, the wavevector is $\mathbf{k}^{(\text{SP})} = (k_x^{(\text{SP})}, k_y^{(\text{SP})}, k_z^{(\text{SP})})$. If we consider spaceplates made up of plates or layers whose interfaces are x, y planes, then $k_x^{(\text{SP})} = k_x^{(\text{BG})}$ and $k_y^{(\text{SP})} = k_y^{(\text{BG})}$ due to momentum conservation. Consequently, such a spaceplate will automatically reproduce the contribution to the phase due to displacement in $\mathbf{r}$ by x and y, *i.e.*, $\phi_{\text{SP}} = k_x^{(\text{SP})} x + k_y^{(\text{SP})} y + k_z^{(\text{SP})} d = k_x^{(\text{BG})} x + k_y^{(\text{BG})} y + k_z^{(\text{SP})} d$. The remaining component to be reproduced is the phase due to the displacement along z in the medium, $\phi_{\text{BG}} = k_z^{(\text{BG})} d_{\text{eff}}$. In a homogeneous spaceplate this phase is $\phi_{\text{SP}} = k_z^{(\text{SP})} d$. With this definition, the goal reduces to making the Fourier transfer function for propagation, $H = \exp(i\phi)$, equal for the spaceplate and the medium up to a global offset phase ϕ_{G}:

$$e^{i\phi_{\text{SP}}} = e^{i\phi_{\text{BG}} + i\phi_{\text{G}}}$$

$$\implies 2\pi m = \phi_{\text{SP}} - \phi_{\text{BG}} - \phi_{\text{G}} \tag{1}$$

$$2\pi m = k_z^{(\text{SP})} d - k_z^{(\text{BG})} d_{\text{eff}} - \phi_{\text{G}}, \tag{2}$$

where m is an integer.

If the plane-wave is traveling in the medium at an angle θ to the z-axis, we can express the z-component of the wavevector as

$$k_z^{(\text{BG})} = k_{\text{BG}} \cos\theta. \tag{3}$$

In order to match this $\cos\theta$ variation, the spaceplate must produce an angle-dependent phase. To produce this dependence, one possible scenario is a spaceplate made of a non-isotropic material. That is, we consider a spaceplate material with a refractive index $n(\theta_{\text{SP}})$ that varies with the wavevector angle inside the spaceplate θ_{SP}, such that the wavenumber would be $k_{\text{SP}} = n(\theta_{\text{SP}})k_0$. In this case, the z-component of the wavevector is

$$k_z^{(\text{SP})} = n(\theta_{\text{SP}})k_0 \cos\theta_{\text{SP}} = \tilde{n} k_{\text{BG}} \cos\theta_{\text{SP}}, \tag{4}$$

where we defined the refractive index ratio, $\tilde{n} = \tilde{n}(\theta_{\text{SP}}) \equiv n(\theta_{\text{SP}})/n_{\text{BG}}$. Since transverse momentum is conserved throughout, we define it as a single parameter $k_\perp \equiv \sqrt{(k_x^{(\text{SP})})^2 + (k_y^{(\text{SP})})^2} = \sqrt{(k_x^{(\text{BG})})^2 + (k_y^{(\text{BG})})^2}$. In particular, by the Pythagorean theorem, $k_\perp^2 = k_{\text{SP}}^2 - \left(k_z^{(\text{SP})}\right)^2$ and, also,

$$\begin{aligned} k_z^{(\text{BG})} &= \sqrt{k_{\text{BG}}^2 - k_\perp^2} \\ &= \sqrt{k_{\text{BG}}^2 - \left(k_{\text{SP}}^2 - \left(k_z^{(\text{SP})}\right)^2\right)} \\ &= \sqrt{k_{\text{BG}}^2 - \tilde{n}^2 k_{\text{BG}}^2 \left(1 - \cos^2\theta_{\text{SP}}\right)} \\ &= k_{\text{BG}} \sqrt{1 - \tilde{n}^2 \sin^2\theta_{\text{SP}}}. \end{aligned} \tag{5}$$

The goal of these manipulations was to ensure that the only angle that appears in the expressions for both wavevectors is θ_{SP}. Note that these relations inherently account for refraction at the interfaces.

We insert the two wavevector equations, Eq. (4) and Eq. (5), into the phase condition in Eq. (1) to obtain,

$$2\pi m = k_{\text{BG}}\, d\, \tilde{n} \cos\theta_{\text{SP}} - k_{\text{BG}}\, d_{\text{eff}} \sqrt{1 - \tilde{n}^2 \sin^2\theta_{\text{SP}}} - \phi_{\text{G}}. \tag{6}$$

We can rearrange this equation to isolate the phase offsets in a single parameter,

$$C \equiv \left(m + \frac{\phi_{\text{G}}}{2\pi}\right) \frac{\lambda}{n_{\text{BG}} d} = \tilde{n} \cos\theta_{\text{SP}} - \frac{d_{\text{eff}}}{d} \sqrt{1 - \tilde{n}^2 \sin^2\theta_{\text{SP}}}. \tag{7}$$

We then solve for $\tilde{n}$ and recall the definition of $\mathcal{R} = d_{\text{eff}}/d$ to yield Eq. (2) in the main text, the general equation that describes the index of refraction for a homogeneous spaceplate,

$$\tilde{n}(\theta_{\text{SP}}) = \frac{n(\theta_{\text{SP}})}{n_{\text{BG}}} = \frac{C \pm \sqrt{C^2 + (\mathcal{R}^2 - C^2)(1 + \mathcal{R}^2 \tan^2\theta_{\text{SP}})}}{(1 + \mathcal{R}^2 \tan^2\theta_{\text{SP}}) \cos\theta_{\text{SP}}}. \tag{8}$$

The physical meaning of C can be understood by re-expressing it as

$$C(\theta_{\text{SP}}) = \frac{2\pi m(\theta_{\text{SP}}) + \phi_{\text{G}}}{k_{\text{BG}} d}, \tag{9}$$

where we have made the potential dependence of the integer m on angle explicit. C is the ratio of the total phase offset to the phase ϕ_{BG} accumulated by a wave at angle $\theta_{\text{SP}} = \theta = 0$, traveling distance d.

4.2 Discussion of specific solutions

An infinite family of solutions are parametrized by Eq. (8). First, there are two branches to the solution corresponding to the positive and negative roots and, second, C is arbitrary. In the following subsections, we describe some of these solutions.

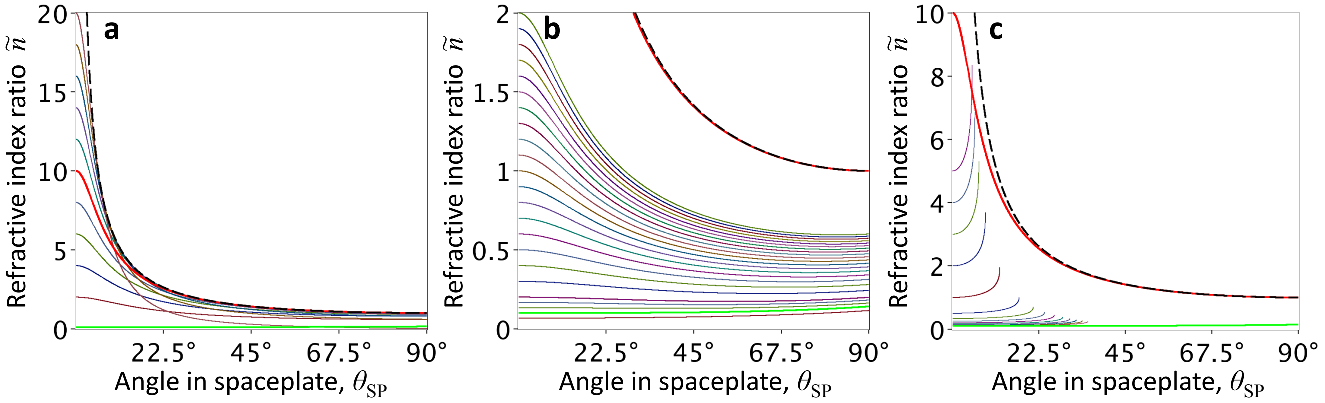

Supplementary Figure 6 | General angle-dependent refractive index solutions for a spaceplate. Plotted is the index ratio, $\tilde{n} \equiv n(\theta_{\mathrm{SP}})/n_{\mathrm{BG}}$. In the three panels, all the solutions are for $\mathcal{R} = 10$, the black dashed line is the bound on all solutions (Eq. (13)), the red line corresponds to a uniaxial spaceplate ($C = 0$, Eq. (12)), and the green line corresponds to the solution approximated by the low-index spaceplate ($C = 1/\mathcal{R} - \mathcal{R}$). **a,** Positive root solutions for $-\mathcal{R} \leq C \leq \mathcal{R}$, *i.e.*, $C = \mathcal{R}(j/5 - 1)$ for $j = 0$ to 20. **b,** Positive root solutions for $C = j/\mathcal{R} - \mathcal{R}$ with $j = 1$ to 10 and $3j = 2$ to 6. **c,** Negative root solutions for $C = \mathcal{R} + j$ with $j = 1$ to 5 and $1/j = 1$ to 10. For every positive-valued refractive index solution $\tilde{n}_+$, there is a mirror negative-valued solution, $\tilde{n}_- = -\tilde{n}_+$.

4.2.1 Uniaxial spaceplate

We start with the positive-root solution. We first consider the $C = 0$ solution. The refractive index ratio described by this solution is

$$\begin{aligned} \tilde{n}(\theta_{\mathrm{SP}}) &= \frac{\pm\sqrt{\mathcal{R}^2(1+\mathcal{R}^2\tan^2\theta_{\mathrm{SP}})}}{(1+\mathcal{R}^2\tan^2\theta_{\mathrm{SP}})\cos\theta_{\mathrm{SP}}} \\ &= \frac{\pm\mathcal{R}}{\cos\theta_{\mathrm{SP}}\sqrt{1+\mathcal{R}^2\tan^2\theta_{\mathrm{SP}}}}. \end{aligned} \tag{10}$$

Taking the positive root and using $\tilde{n} = n(\theta_{\mathrm{SP}})/n_{\mathrm{BG}}$, Eq. (10) can be re-expressed in the standard form for the extraordinary index of a birefringent uniaxial crystal:

$$\begin{aligned} \frac{1}{n^2(\theta_{\mathrm{SP}})} &= \frac{\cos^2\theta_{\mathrm{SP}}\left(1+\mathcal{R}^2\tan^2\theta_{\mathrm{SP}}\right)}{n_{\mathrm{BG}}^2\mathcal{R}^2} \\ &= \frac{\cos^2\theta_{\mathrm{SP}}}{n_{\mathrm{BG}}^2\mathcal{R}^2} + \frac{\sin^2\theta_{\mathrm{SP}}}{n_{\mathrm{BG}}^2} \\ &= \frac{\cos^2\theta_{\mathrm{SP}}}{n_{\mathrm{o}}^2} + \frac{\sin^2\theta_{\mathrm{SP}}}{n_{\mathrm{e}}^2}. \end{aligned} \tag{11}$$

The last line is valid if the crystal has its extraordinary axis along z, is negative uniaxial ($n_{\mathrm{o}} > n_{\mathrm{e}}$), $n_{\mathrm{BG}} = n_{\mathrm{e}}$, and $\mathcal{R} = n_{\mathrm{o}}/n_{\mathrm{e}}$. In this case, it will impart the ideal angle-dependent phase to mimic $d_{\mathrm{eff}} = \mathcal{R}d$ of propagation in a medium with n_{BG}.

Since this solution has no global phase offset ($\phi_{\mathrm{G}} = 0$), not only will it act as a spaceplate for imaging, it will also replace d_{eff} in an interferometer. This will generally not be true; Consider, for example, an interferometer situated in a vacuum background ($n_{\mathrm{BG}} = 1$). A glass plate of index $n = 1.5$ and thickness

d can replace the 'optical path length' $d_{\text{eff}} = 1.5d$ of interferometer arm length. Contrast this with the action of the same glass plate on the focus location of a beam propagating in vacuum. Counter to the situation in the the interferometer, refraction at the plate boundaries makes the focus shift further along the propagation direction, as if it had passed through distance $d_{\text{eff}} = (n_{\text{BG}}/n)d = (d/1.5)$ in vacuum[2]. This is the opposite effect to what we seek, showing that the established concept of optical path length is not typically appropriate for a spaceplate. However, since the global phase offset is zero in this case, this uniaxial spaceplate will mimic propagation for the purpose of imaging and interferometry.

4.2.2 Low-index spaceplate

In the family defined by $C > (1-\mathcal{R})$, $n(\theta_{\text{SP}})$ is globally smaller than the refractive index of the background medium, *i.e.,* $n(\theta_{\text{SP}}) < n_{\text{BG}}$. The special case where $C = 1/\mathcal{R} - \mathcal{R}$ exhibits the lowest curvature at $\theta_{\text{SP}} = 0$ in this family. In other words, it is the flattest solution for small angles and, thus, has the lowest dependence on angle. Consequently, an approximation to this solution is a medium with no angular dependence at all, an isotropic medium with a refractive index $n(0) \equiv n$. For this low-index spaceplate, $\mathcal{R} = (n_{\text{BG}}/n)$.

4.2.3 Other solutions

Many other solutions are possible. For each value of $C \equiv C_+$, there is a positive root solution $\tilde{n}_+$, *i.e.,* taking the $+$ sign in Eq. (8). (Note, C_+ can be positive or negative.) Paired with this positive root solution there is a mirror negative root solution $\tilde{n}_-$, with $C = -C_+$. It is a mirrored about the $\tilde{n} = 0$ line, in that the indices have the same magnitude but opposite sign, $\tilde{n}_- = -\tilde{n}_+$. A family of negative root solutions will have a refractive index that is positive for all angles, $\tilde{n}_-(\theta_{\text{SP}}) > 0$. We plot some of these solutions in Supp. Fig. 6c. Critically, it is unknown which index profiles $\tilde{n}(\theta_{\text{SP}})$ are physically allowable by Maxwell's equations.

4.2.4 General properties of the solutions

We now consider some limiting cases for the angle-dependent spaceplate refractive index. We first consider a limit in which both C and $\mathcal{R}$ become large. That is, we take $C' = tC$ and $\mathcal{R}' = t\mathcal{R}$ and take the large t limit:

$$\lim_{t\to\infty} \tilde{n}\left(\theta_{\text{SP}}\right) = \frac{\pm\sqrt{1-\frac{C'^2}{\mathcal{R}'^2}}}{|\sin\theta_{\text{SP}}|}. \tag{12}$$

Consequently, in this limit, all the solutions have the same simple angular dependence up to an overall scaling factor.

Moreover, all the solutions, regardless of C and $\mathcal{R}$, will fall between these two curves:

$$\lim_{\mathcal{R}\to\infty} \tilde{n}\left(\theta_{\text{SP}}\right) = \frac{\pm 1}{|\sin\theta_{\text{SP}}|}, \tag{13}$$

indicated by the black dashed curves in Supp. Fig. 6. The compression factor and C parameter can be expressed in terms of the value of $\tilde{n}$ at two angles. First, at normal incidence,

$$\tilde{n}\left(\theta_{\text{SP}} = 0^\circ\right) = C \pm \mathcal{R}. \tag{14}$$

Second, while not all solutions give a real-valued refractive index ratio out to $\theta_{\text{SP}} = 90^\circ$, for those that do,

$$\tilde{n}\left(90^\circ\right) = \pm\sqrt{1-\frac{C^2}{\mathcal{R}^2}}. \tag{15}$$

Consequently, in terms of these two boundaries values of $\tilde{n}$, the compression factor is given by,

$$\mathcal{R} = \pm\tilde{n}(0^\circ)\left(1 \pm \sqrt{1 - \frac{1}{(\tilde{n}(90^\circ))^2}}\right). \tag{16}$$

5 Low-index spaceplate measurements

Supplementary Section 4.2.2 introduced the solution to Eq. 2 in the main text for which $C = (1/\mathcal{R} - \mathcal{R})$. This solution describes a spaceplate index that is lower than that of the background medium for all angles, $n(\theta_{SP}) < n_{BG}$. It is also the flattest of any solution near $\theta_{SP} = 0$ and, thus, corresponds to an approximately isotropic medium $n(\theta_{SP}) \equiv n_{LI}$, particularly for small incident angles. For this low-index spaceplate, $\mathcal{R} = (n_{BG}/n_{LI})$.

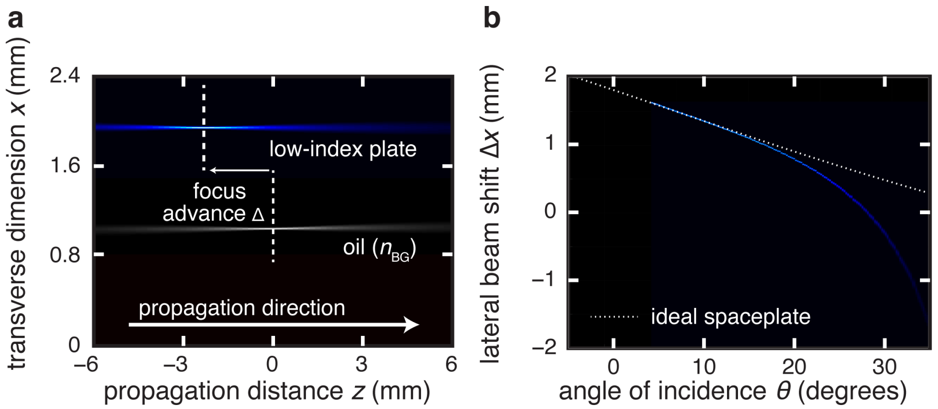

Supplementary Figure 7 | Space compression with a low-index spaceplate. For all plots, the false-colour along the plot-vertical gives the transverse intensity distribution along x at each z distance on the horizontal plot axis, with paler colour corresponding to higher intensity. **a,** Focal shift, $\Delta = d - d_{\text{eff}}$. Bottom data: Oil (grey). A converging beam comes to focus in oil at $z = 0$. Top data: Low-index spaceplate (blue). Propagation through a plate of air advances the focus position along z by $\Delta = -2.3$ mm. The corresponding y intensity distributions are shown in Supplementary Note 6.1, demonstrating a fully two-dimensional advance. **b,** The walk-off of a beam incident at an angle θ. The dashed line give the lateral beam shift for an ideal spaceplate (*i.e.,* $\Delta x = -(\mathcal{R} - 1)d\sin\theta$) with the same thickness d and compression factor $\mathcal{R}$ as the spaceplate in **(a)**. Above an incident angle of $\theta = 15^\circ$, the low-index spaceplate starts to exhibit noticeable aberrations, deviating from the dotted line, due to the onset of total internal reflection and the failure of the small-angle approximation.

If the background medium is vacuum, then n_{LI} must be less than one, a seemingly unusual property. Nonetheless, there exist both natural and metamaterials (*e.g.,* epsilon-near-zero (ENZ) materials) from which such a spaceplate can be made[3,4]. Current low-index ($n < 1$) materials are prohibitively lossy[3,4], so instead of vacuum, we select a background medium with a higher refractive index, linseed oil (which has $n_{BG} = 1.48$), and use air as the low-index medium. The low-index plate is a $d = 4.4$-mm-long cylinder containing air and faced with glass coverslips. With air as the low index medium ($n_{LI} = 1$), the resulting compression factor is $\mathcal{R} = 1.48$. We perform the same beam focus advance measurement as for the calcite spaceplate in the main text (as described under Beam Measurements in Sec. 1). The measured shift $\Delta = -2.3$ mm agrees well with the predicted shift of $\Delta = (1 - \mathcal{R})d = -2.1$ mm.

We next experimentally investigate the transverse displacement of a beam incident on the low-index spaceplate by rotating it with respect to the incoming beam. We see that for larger angles, the displacement induced by the low-index plate deviates from that of an ideal spaceplate (see Sec. 9: Transverse and lateral beam shifts due to a spaceplate). In an imaging system, this discrepancy would act to introduce optical aberrations. This deviation is due to the failure of the small-angle approximation, most dramatically near the onset of total internal reflection at $\theta_{\mathrm{crit}} = 42.5°$.

Aside from this aberration, total internal reflection imposes a severe limitation on the low-index spaceplate; as its refractive index n_{LI} decreases, its acceptance angle decreases as $\theta_{\mathrm{crit}} = \arcsin(n_{\mathrm{LI}}/n_{\mathrm{BG}}) = \arcsin(1/\mathcal{R})$. Consequently, for the low-index spaceplate, the greater the compression factor $\mathcal{R}$ is, the smaller the numerical aperture (NA, *i.e.,* θ_{crit}) will be.

This measurement demonstrates a polarization-independent spaceplate effect for a slightly larger compression ($\mathcal{R} = 1.48$). More importantly, it further validates our theory and Eqs. 1 – 2 in the main text.

6 Polarization measurements

In this section, we present measurements on polarized beams with the uniaxial spaceplates. Using the polarization control detailed in Section 1, we repeat the beam measurements with an ordinarily polarized beam. While for extraordinarily polarized light the uniaxial crystal acts as a spaceplate, for ordinarily polarized light it acts a homogeneous isotropic medium with refractive index $n_{\mathrm{o}} > n_{\mathrm{BG}}$. Consequently, it acts in the opposite manner to a spaceplate; this must be compensated with more propagation distance in a given setup. We measured the uniaxial crystal's effect on the focal shift of an ordinarily polarized beam and, also, on the beam's lateral shift when the spaceplate is tilted. The experimental results are shown in

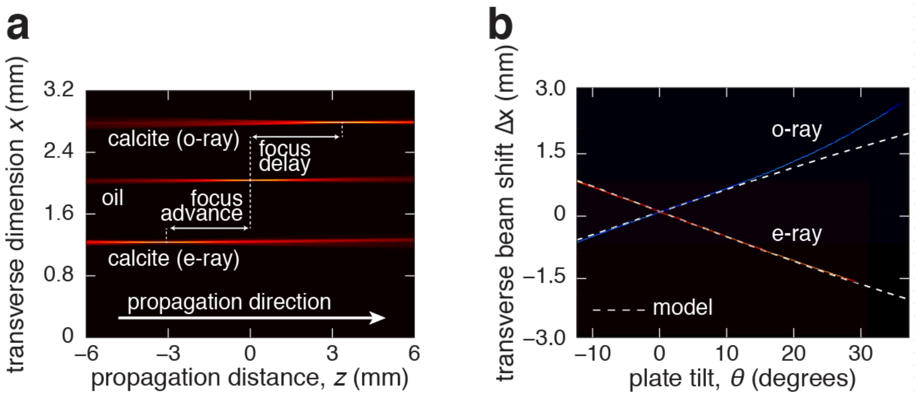

Supplementary Figure 8 | Polarized beam measurements with the uniaxial spaceplate. With an extraordinarily (e-ray) polarized beam, we repeated the calcite measurements reported in Fig. 3 (see the main paper for details). For comparison, we also include the results for an ordinarily (o-ray) polarized beam. **a,** Focal shift Δ along z. Relative to the focus location in the absence of a spaceplate (oil), the o-ray has its focus shifted further from the spaceplate, *i.e.,* delayed. The spaceplate effect is evident in the e-ray, which has its focus advanced towards the spaceplate. **b,** The lateral shift Δx of a beam due to tilting the uniaxial spaceplate by θ relative to the z-axis. The o-ray shifts in opposite manner to what is required by a spaceplate, in contrast to the e-ray. The dashed lines are Δx for an ideal spaceplate with (e-ray) compression factor $\mathcal{R} = n_{\mathrm{o}}/n_{\mathrm{e}} = 1.117$ matching calcite and (o-ray) $\mathcal{R} = n_{\mathrm{BG}}/n_{\mathrm{o}} = 0.895$. Notice that the lateral shift of the o-ray deviates from the approximately straight theory line whereas the e-ray does not. This deviation is evidence of the aberrations that ordinarily polarized light will encounter. In contrast, the uniaxial crystal operates ideally for all angles.

Supp. Fig. 8. The focal shift observed for the ordinarily polarized beam was $\Delta = 3.2$ mm away from the crystal which agrees well with the theoretical shift $\Delta = -(\mathcal{R}-1)d = 3.126$ mm for $\mathcal{R} = n_{\mathrm{BG}}/n_{\mathrm{o}} = 0.895$.

6.1 Evidence for a fully two-dimensional spaceplate effect

All three spaceplate types that we have introduced function in both transverse directions, x and y. That is, the spaceplate advances the propagation of the full two-dimensional spatial distribution of the light-field. To demonstrate this, in this section, we present the same measurement as done in Fig. 3a but now projecting the beam along y. Consequently, the plot-vertical gives the intensity distribution along y.

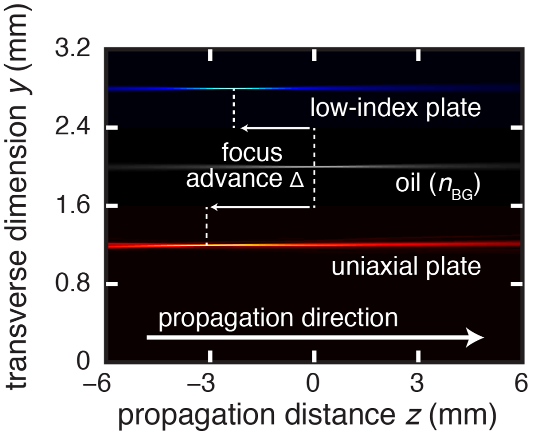

Supplementary Figure 9 | Experimental demonstration of space compression. The colour along the plot-vertical gives the transverse intensity distribution along y at each point along the horizontal plot axis for all plots. Focal shift, $\Delta = d - d_{\mathrm{eff}}$. Middle data: Oil (grey). A converging beam comes to focus in oil at $z = 0$. Top data: Low-index spaceplate (blue). Propagation through a plate of air advances the focus position along z by $\Delta = -2.3$ mm. Bottom data: Uniaxial spaceplate (red). Propagation of an extraordinary polarized beam through a calcite crystal with its fast axis along z advances the focus position by $\Delta = -3.4$ mm.

7 Implications of causality

Causality does not appear to limit the operation of a possible spaceplates with regards to the maximum achievable compression factors $\mathcal{R}$. When extrapolating from the demonstrations above, it is evident that a spaceplate can be designed to exhibit an arbitrarily large $\mathcal{R}$ for a single operating frequency by employing a medium with a sufficiently low refractive index. Instead, this system reveals an explicit trade-off between $\mathcal{R}$ and the numerical aperture of the spaceplate ($\mathrm{NA} = (1/\mathcal{R})$). However, whether this trade-off is inherent to the spaceplate concept or is a consequence of the low-index spaceplate implementation remains an open question. A broadband spaceplate based on an index less than unity would exhibit a violation of microscopic causality, an energy velocity at a point inside the medium that is higher than c. Thus, the bandwidth of a low-index spaceplate will also be constrained.

In contrast, a uniaxial spaceplate designed to work in vacuum ($n_{\mathrm{e}} = 1$, $n_{\mathrm{o}} = \mathcal{R}$) will not violate microscopic causality; its operation depends on the ratio of its two refractive indices, and these two quantities are not causally related. To this point, the group index in a non-dispersive uniaxial material is always bounded by its birefringent indices, n_{e} and n_{o}, and, thus, the group velocity will always be less than c. Indeed, our experimental demonstration supports the prospect of full broadband operation of a spaceplate. Microscopic causality also does not exclude the possibility of spaceplate operation in free space that is based on anisotropic materials.

This conclusion may seem to conflict with intuition derived from experience with other transformation optics-based devices[5], such as an invisibility cloak. A functioning cloak necessitates light travelling within a medium to keep pace with light propagating in the surrounding background. This restriction on macroscopic causality may be relaxed by immersing the device within a high-index medium since then light propagating in the surrounding medium will be slowed. By contrast, a spaceplate encompasses the entire imaging beam, and it has no physical boundary in the direction transverse to the direction of propagation to impose such a strict condition. Therefore, there is no penalty for a beam propagating through a spaceplate to be delayed with respect to another beam propagating in free space alongside it. Consequently, the overall time delay for the imaging light to pass through the spaceplate is unconstrained, becoming a free parameter in spaceplate design. For this reason, we do not foresee any intrinsic issues with limited operation bandwidth in the spaceplate platform, despite evidence to the contrary in other systems.

We suspect that rigorous bandwidth limits could be derived for specific implementations of spaceplates using methods that have already been applied to metasurfaces (*e.g.,* those described in Ref.[6]). These investigations are of interest for future research.

If causality was ultimately revealed to pose a restriction on broadband operation, a spaceplate operating at a single frequency would still be beneficial towards many applications. For instance, there is a great deal of interest in wavefront shaping applications that make use of the transverse degree of freedom of light, such as spatial multiplexing in telecommunications[7], or high-dimensional quantum cryptography[8]. Applications of these type notably operate at a single frequency and could usually benefit from being compressed by a spaceplate. Additionally, as is argued in the main text, many imaging applications only use three colours centered around red, green, and blue frequencies to enable full-colour imaging. A spaceplate that can perform simultaneous compressions for three discrete frequencies could enable full-color imaging without the need of being broadband. The question of broadband operation or causality can, fortunately, be circumvented for these applications.

8 Fabricated spaceplates

The low-index spaceplate was fabricated by attaching microscope cover slips to both ends of a lens tube using epoxy (Supp. Fig. 10a). The uniaxial spaceplate was fabricated by a commercial vendor, which informed us that, in calcite, surfaces normal to the extraordinary axis are difficult to polish well (Supp. Fig. 10b). Consequently, some roughness can be observed on the entrance and exit surfaces, resulting in scattering, particularly in the beam measurements.

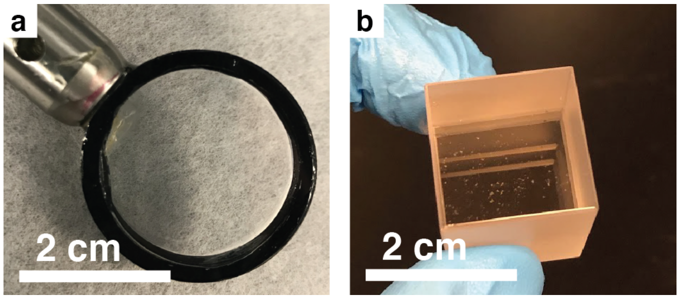

Supplementary Figure 10 | Fabricated spaceplates. **(A)** Low-index spaceplate. **b,** Uniaxial spaceplate. The entrance and exit surfaces exhibit visible roughness.

9 Transverse and lateral beam shifts due to a spaceplate

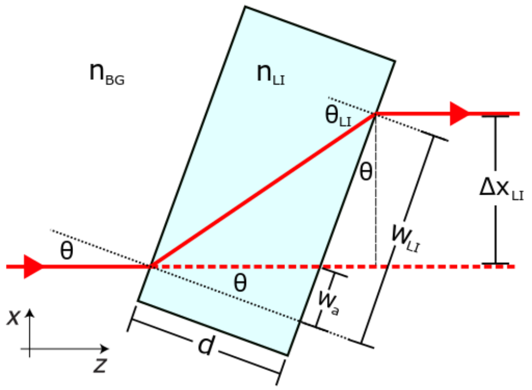

Supplementary Figure 11 | Geometry for the derivation of lateral beam shift Δx_{LI} in the case of a low-index plate.

As depicted in Fig. 1a in the main text, one expects a spaceplate to shift a beam of light when incident at an angle θ. In this section, we derive the transverse shift w and lateral shift Δx. We first derive the shift due to propagating through a slab of medium n_{BG} of thickness d_{eff}, which defines the effect of an ideal spaceplate. We then derive these shifts for the low-index spaceplate and uniaxial spaceplate.

9.1 Beam shifts due to an ideal spaceplate

Consider a beam traveling in the x,z plane at an angle θ to the z-axis. The beam traverses a region (*i.e.,* a slab) of a medium at length $z = d_{\mathrm{eff}}$, entering at point $(x_{\mathrm{in}}, z = 0)$ and exiting at point $(x_{\mathrm{out}}, z = d_{\mathrm{eff}})$. Upon exiting the region the beam is parallel to the beam that entered the region. That is, its angle is unchanged.

By simple geometry the beam shifts in the x-direction by an amount,

$$x_{\mathrm{out}} - x_{\mathrm{in}} = -d_{\mathrm{eff}} \tan\theta \equiv w, \tag{17}$$

which we name the ‘transverse’ shift. The negative sign is due to a convention that will be explained in the next paragraph. For an ideal spaceplate, the same beam will exit translated along the plate’s interface by an equal amount w of transverse shift as created by the slab of medium described above. Another way of looking at the transverse shift is as a displacement from a line that both goes through point $(x_{\mathrm{in}}, z = 0)$ and is normal to the plate interfaces. In contrast to the scenario with the medium, this shift now occurs at a distance $z = d$, the plate thickness.

To test this effect, rather than tilt the incoming beam by θ, it is experimentally simpler to tilt the studied spaceplate by the same angle. In this case, the quantity that can be measured most directly is the ‘lateral’ shift Δx of the beam. This is the shift of the beam along an axis normal to the beam’s propagation direction. The sign convention mentioned in the last paragraph sets Δx to be positive if the lateral shift is below the path of the incident beam, as depicted in Supp. Fig. 11. In particular, Δx would be positive for a beam passing through a tilted regular glass plate in a vacuum background medium.

To relate Δx to w, one must account for the tilt of the normal of the plate interface. This alone would result in apparent transverse shift of $w_a = d \tan\theta$, which must be added to the actual transverse shift w.

With this established, geometry gives the lateral shift as,

$$\begin{aligned}\Delta x &= (w + w_a)\cos\theta \\ &= (-d_{\text{eff}}\tan\theta + d\tan\theta)\cos\theta \\ &= -d\,(\mathcal{R}-1)\sin\theta,\end{aligned} \tag{18}$$

where we have used $d_{\text{eff}} = \mathcal{R}d$.

9.2 Beam shifts due to a low-index spaceplate

We now consider the lateral shift created by a plate made of an isotropic and homogeneous medium of thickness d. If the medium's refractive index $n_{\text{LI}} < n_{\text{BG}}$, then the plate is a low-index spaceplate. To start, however, we leave n_{LI} unrestricted. At the entrance interface, incoming light refracts to angle θ_{LI} inside the plate according to Snell's law. In the following, we use Snell's law to derive analogous relations for cos and tan:

$$\begin{aligned}\sin\theta_{\text{LI}} &= \frac{n_{\text{BG}}}{n_{\text{LI}}}\sin\theta, \\ \cos\theta_{\text{LI}} &= \sqrt{1-\sin^2\theta_{\text{LI}}} = \sqrt{1-\left(\frac{n_{\text{BG}}}{n_{\text{LI}}}\right)^2\sin^2\theta}, \\ \tan\theta_{\text{LI}} &= \frac{\sin\theta_{\text{LI}}}{\cos\theta_{\text{LI}}} = \frac{\frac{n_{\text{BG}}}{n_{\text{LI}}}\sin\theta}{\sqrt{1-\left(\frac{n_{\text{BG}}}{n_{\text{LI}}}\right)^2\sin^2\theta}} = \frac{\sin\theta}{\sqrt{\left(\frac{n_{\text{LI}}}{n_{\text{BG}}}\right)^2-\sin^2\theta}}.\end{aligned} \tag{19}$$

As in Eq. (17) from the last section, simple geometry shows that the transverse shift of the beam is,

$$w_{\text{LI}} = -d\tan\theta_{\text{LI}}. \tag{20}$$

Likewise, the lateral shift is,

$$\begin{aligned}\Delta x_{\text{LI}} &= (w_{\text{LI}} + w_a)\cos\theta \\ &= (-d\tan\theta_{\text{LI}} + d\tan\theta)\cos\theta.\end{aligned} \tag{21}$$

Substituting Eq. (19) into Eq. (21) yields an expression for the lateral shift that is dependent only on θ,

$$\Delta x_{\text{LI}} = -d\sin\theta\left(\frac{\cos\theta}{\sqrt{\left(\frac{n_{\text{LI}}}{n_{\text{BG}}}\right)^2-\sin^2\theta}}-1\right). \tag{22}$$

This shows that the lateral shift created by a low-index spaceplate is not equal to the ideal lateral shift given in Eq. (18). However, using the small angle approximation ($\theta \ll 1$) we find that

$$\begin{aligned}\Delta x_{\text{LI}} &\approx -d\sin\theta\left(\frac{1}{\sqrt{\left(\frac{n_{\text{LI}}}{n_{\text{BG}}}\right)^2-0}}-1\right)+O\left(\theta^3\right) \\ &= -d\sin\theta\left(\left|\frac{n_{\text{BG}}}{n_{\text{LI}}}\right|-1\right) \\ &= -d\,(\mathcal{R}-1)\sin\theta \\ &= \Delta x,\end{aligned} \tag{23}$$

where we have identified the low-index compression factor, $\mathcal{R} = |n_{\mathrm{BG}}/n_{\mathrm{LI}}|$. For large angles, the low-index spaceplate will produce an incorrect shift, effectively introducing aberrations. Moreover, all angles above the critical angle, $\theta_c = \arcsin(n_{\mathrm{LI}}/n_{\mathrm{BG}})$, are perfectly reflected. However, for small angles a low-index plate acts as an ideal spaceplate, *i.e.,* $\Delta x_{\mathrm{LI}} = \Delta x$.

While we have derived the lateral shift Δx_{LI} in the context of a low-index spaceplate, it is actually valid for any value of n_{LI}, including a negative refractive index. Moreover, we use it to describe the action of the uniaxial spaceplate on ordinarily polarized light, which effectively experiences a homogeneous isotropic medium with index n_{o}. Lastly, the shift Δx_{LI} also shows that a simple glass plate in air will introduce imaging aberrations for large angles.

9.3 Beam shifts due to a uniaxial spaceplate

We will now derive the lateral shift created by a uniaxial spaceplate. The uniaxial crystal is characterized by two indices, the extraordinary and ordinary refractive indices, n_{e} and n_{o}, respectively. To derive the shift, one must account for the anomalous refraction and consequent beam walk-off that generally occurs with a birefringent medium. In particular, for extraordinarily polarized light in a uniaxial crystal (U), the Poynting vector $\mathbf{S}^{(\mathrm{U})}$, which describes the energy flow, can point in a direction different from the wavevector $\mathbf{k}^{(\mathrm{U})}$. In contrast, an ordinarily polarized beam will refract normally (*i.e.,* the Poynting and wavevectors are parallel), and, hence, will shift according to Eq. (23) but with n_{LI} replaced with n_{o}. In the following, we use the results from Ref. [9], which carefully analyzed the Poynting vector angle at the interface between a homogeneous medium and a uniaxial crystal with its extraordinary axis (*i.e.,* optic axis) at an arbitrary angle. Since the Poynting vector is the direction an extraordinary beam travels along in the crystal, one can use it to follow a geometric derivation similar to that used in Eq. (18).

As discussed earlier in the SI, the transverse component of the wavevector is conserved across the interface,

$$k_{\perp}^{(\mathrm{U})} = k_{\perp}^{(\mathrm{BG})} \equiv k_{\perp} = k_0 n_{\mathrm{BG}} \sin\theta. \tag{24}$$

We apply the results from Ref. [9] to the case where the extraordinary axis is normal to the plate interface. In this case, the z-component of the wavevector in the crystal can be expressed as:

$$k_z^{(\mathrm{U})} = n_{\mathrm{o}} k_0 \sqrt{1 - \left(\frac{n_{\mathrm{BG}}}{n_{\mathrm{e}}}\right)^2 \sin^2\theta}. \tag{25}$$

If one considers the case where $n_{\mathrm{BG}} = n_{\mathrm{e}}$, then $k_z^{(\mathrm{U})} = n_{\mathrm{o}} k_0 \cos\theta$, which directly shows why a uniaxial crystal acts as a spaceplate: it produces an angle-dependent phase that is magnified by a factor $n_{\mathrm{o}}/n_{\mathrm{e}}$ relative to propagation through a medium with $n_{\mathrm{BG}} = n_{\mathrm{e}}$. For now, however, we leave n_{e} general.

Whereas, after refraction at the interface, the wavevector angle in the crystal will be given by $\tan\theta_{\mathrm{U},k} = k_{\perp}^{(\mathrm{U})}/k_z^{(\mathrm{U})}$, Ref. [9] showed that the Poynting vector of an extraordinarily polarized plane-wave will be along a potentially different angle,

$$\begin{aligned} \tan\theta_{\mathrm{U},S} &= \frac{n_{\mathrm{o}}^2 k_{\perp}^{(\mathrm{U})}}{n_{\mathrm{e}}^2 k_z^{(\mathrm{U})}} \\ &= \frac{n_{\mathrm{o}}^2 k_0 n_{\mathrm{BG}} \sin\theta}{n_{\mathrm{e}}^2 n_{\mathrm{o}} k_0 \sqrt{1 - \left(\frac{n_{\mathrm{BG}}}{n_{\mathrm{e}}}\right)^2 \sin^2\theta}}, \end{aligned}$$

where we have used the expressions for the two wavevector components, Eqs. (24) and (25).

From here on, we restrict ourselves to the case in which $n_{\mathrm{BG}} = n_{\mathrm{e}}$. In this case, the Poynting vector angle reduces to

$$\tan\theta_{\mathrm{U},S} = \mathcal{R}\tan\theta, \tag{26}$$

where we have identified the compression factor as $\mathcal{R} = n_{\mathrm{o}}/n_{\mathrm{e}}$, as expected from our phase analysis earlier. Using Eq. (26), the transverse shift for an extraordinarily polarized beam will be

$$w_{\mathrm{U}} = -d\mathcal{R}\tan\theta, \tag{27}$$

which can be used to find the associated lateral shift:

$$\begin{aligned} \Delta x_{\mathrm{U}} &= (w_{\mathrm{U}} + w_a)\cos\theta \\ &= \left(-d\tan\theta_{\mathrm{U},S} + d\tan\theta\right)\cos\theta \\ &= -d\,(\mathcal{R}-1)\sin\theta \\ &= \Delta x. \end{aligned} \tag{28}$$

This shows that for all angles, the lateral shift of an extraordinarily polarized beam Δx_{U} will be identical to the lateral shift of an ideal spaceplate Δx. In this sense, a uniaxial crystal acts as a perfect spaceplate for the purposes of imaging, replacing medium n_{BG}, while introducing no aberrations.

Supplementary References

1. Kingslake, R. *Lens design fundamentals* (Academic Press, 2010), second edn.

2. Hobbs, P. C. D. *Building electro-optical systems: Making it all work* (John Wiley & Sons, Inc., Hoboken, NJ, USA, 2009), second edn.

3. Engheta, N. Pursuing near-zero response. *Science* **340**, 286–287 (2013).

4. Vulis, D. I., Reshef, O., Camayd-Muñoz, P. & Mazur, E. Manipulating the flow of light using Dirac-cone zero-index metamaterials. *Reports on Progress in Physics* **82**, 012001 (2019).

5. Monticone, F. & Alu, A. Do Cloaked Objects Really Scatter Less? *Physical Review X* **3**, 041005 (2013).

6. Presutti, F. & Monticone, F. Focusing on bandwidth: achromatic metalens limits. *Optica* **7**, 624 (2020).

7. Bozinovic, N. *et al.* Terabit-scale Orbital Angular Momentum Mode Division Multiplexing in Fibers. *Science* **340**, 1545–1548 (2013).

8. Sit, A. *et al.* High-dimensional intracity quantum cryptography with structured photons. *Optica* **4**, 1006 (2017).

9. Entezar, S. R. & Habil, M. K. Refraction and reflection from the interface of anisotropic materials. *Physica Scripta* **94**, 085502 (2019).
