# Supplementary material for: An optic to replace space and its application towards ultra-thin imaging systems
Source: Nat Commun. 2021 Jun 10;12:3512. doi: 10.1038/s41467-021-23358-8 (PMC8192919; doi:10.1038/s41467-021-23358-8)
Supplement: Supplementary file 2 — Description of Additional Supplementary Files [file 41467_2021_23358_MOESM2_ESM.docx]

**Description of Additional Supplementary Files**

**Supplementary Movie 1:** Propagation of a beam in both oil and the air plate as a function of beam propagation distance in the oil z. The green laser beam looks red due to post-processing. This video was integrated to produce Supp. Fig. S7.

**Supplementary Movie 2:** Propagation of a beam in both oil and calcite as a function of beam propagation distance in the oil z. The green laser beam looks red due to post-processing. This video was integrated to produce Fig. 3.

**Supplementary Movie 3:** Propagation of a white-light-illuminated image in both glycerol and calcite as a function of beam propagation distance in the glycerol z.
